# Genomic imbalances defining novel intellectual disability associated *loci*

**DOI:** 10.1186/s13023-019-1135-0

**Published:** 2019-07-05

**Authors:** Fátima Lopes, Fátima Torres, Gabriela Soares, Mafalda Barbosa, João Silva, Frederico Duque, Miguel Rocha, Joaquim Sá, Guiomar Oliveira, Maria João Sá, Teresa Temudo, Susana Sousa, Carla Marques, Sofia Lopes, Catarina Gomes, Gisela Barros, Arminda Jorge, Felisbela Rocha, Cecília Martins, Sandra Mesquita, Susana Loureiro, Elisa Maria Cardoso, Maria José Cálix, Andreia Dias, Cristina Martins, Céu R. Mota, Diana Antunes, Juliette Dupont, Sara Figueiredo, Sónia Figueiroa, Susana Gama-de-Sousa, Sara Cruz, Adriana Sampaio, Paul Eijk, Marjan M. Weiss, Bauke Ylstra, Paula Rendeiro, Purificação Tavares, Margarida Reis-Lima, Jorge Pinto-Basto, Ana Maria Fortuna, Patrícia Maciel

**Affiliations:** 1 Life and Health Sciences Research Institute (ICVS), School of Medicine, University of Minho, 4710-057 Braga, Portugal; 2 ICVS/3B’s - PT Government Associate Laboratory, Braga/Guimarães, Portugal; 3 CGC Genetics, Porto, Portugal; 4 Institute of Biomedical Sciences Abel Salazar (ICBAS), University of Porto, Porto, Portugal; 5 Center for Medical Genetics Dr. Jacinto Magalhães, Porto Hospital Center, Praça Pedro Nunes, Porto, Portugal; 6 Unit for Multidisciplinary Research in Biomedicine, Institute of Biomedical Sciences Abel Salazar (ICBAS), University of Porto, Porto, Portugal; 7 The Mindich Child Health & Development Institute and the Department of Genetics & Genomic Sciences, Icahn School of Medicine at Mount Sinai, New York, NY USA; 8 The Seaver Autism Center for Research and Treatment, Icahn School of Medicine at Mount Sinai, New York, NY USA; 9 Graduate School of Biomedical Sciences, Icahn School of Medicine at Mount Sinai, New York, NY USA; 10 Centro de Genética Preditiva e Preventiva - CGPP, Instituto de Biologia Molecular e Celular - IBMC, Universidade do Porto, Porto, Portugal; 11 Instituto de Investigação e Inovação em Saúde - i3S, Universidade do Porto, Porto, Portugal; 12 Unidade de Neurodesenvolvimento e Autismo do Serviço do Centro de Desenvolvimento da Criança and Centro de Investigação e Formação Clínica, Pediatric Hospital, Centro Hospitalar e Universitário de Coimbra, 3041-80 Coimbra, Portugal; 13 University Clinic of Pediatrics and Institute for Biomedical Imaging and Life Science, Faculty of Medicine, University of Coimbra, Coimbra, Portugal; 14 Medical Genetics Unit, Hospital de Braga, Braga, Portugal; 15 Department of Medical Genetics, Hospital de Faro, Faro, Portugal; 16 Pediatric Neurology Department, Centro Materno-Infantil Centro Hospitalar do Porto, Porto, Portugal; 17 Development Unit, Pediatrics Service, Hospital Centre of Cova da Beira, Covilhã, Portugal; 18 CICS - Health Sciences Research Centre, University of Beira Interior, Covilhã, Portugal; 19 Department of Pediatrics, Médio Ave Hospital Center, Vila Nova de Famalicão, Portugal; 20 Development Unit, Pediatrics Service, Hospital Centre of Cova da Beira, Covilhã, Portugal; 21 Department of Pediatrics, Hospital S. Teotónio, Tondela/Viseu Hospital Center, Viseu, Portugal; 22 Neuropaediatric Unit – Garcia de Orta Hospital, Almada, Portugal; 23 Pediatric and Neonatal Intensive Care, Department of Pediatrics, Porto Hospital Center, Porto, Portugal; 24 Department of Genetics, Hospital D. Estefânia, Lisboa-Norte Hospital Center, Lisbon, Portugal; 25 Genetics Service, Paediatric Department, University Hospital Santa Maria, Lisbon, Portugal; 26 Department of Pediatrics, Médio Ave Hospital Center, Santo Tirso, Portugal; 27 Division of Pediatric Neurology, Department of Child and Adolescent, Centro Hospitalar do Porto e Hospital de Santo António, Porto, Portugal; 28 Neuropsychophysiology Lab, CIPsi, School of Psychology, University of Minho, Braga, Portugal; 29 Department of Pathology, VU University Medical Center, Amsterdam, 1007 MB The Netherlands; 30 Department of Clinical Genetics, VU University Medical Center, Amsterdam, 1007 MB The Netherlands; 31 GDPN- SYNLAB, Porto, Portugal

**Keywords:** CNVs, Neurodevelopment, Genotype-phenotype correlation, *CUL4B* overexpression

## Abstract

**Background:**

High resolution genome-wide copy number analysis, routinely used in clinical diagnosis for several years, retrieves new and extremely rare copy number variations (CNVs) that provide novel candidate genes contributing to disease etiology. The aim of this work was to identify novel genetic causes of neurodevelopmental disease, inferred from CNVs detected by array comparative hybridization (aCGH), in a cohort of 325 Portuguese patients with intellectual disability (ID).

**Results:**

We have detected CNVs in 30.1% of the patients, of which 5.2% corresponded to novel likely pathogenic CNVs. For these 11 rare CNVs (which encompass novel ID candidate genes), we identified those most likely to be relevant, and established genotype-phenotype correlations based on detailed clinical assessment. In the case of duplications, we performed expression analysis to assess the impact of the rearrangement. Interestingly, these novel candidate genes belong to known ID-related pathways. Within the 8% of patients with CNVs in known pathogenic *loci,* the majority had a clinical presentation fitting the phenotype(s) described in the literature, with a few interesting exceptions that are discussed.

**Conclusions:**

Identification of such rare CNVs (some of which reported for the first time in ID patients/families) contributes to our understanding of the etiology of ID and for the ever-improving diagnosis of this group of patients.

**Electronic supplementary material:**

The online version of this article (10.1186/s13023-019-1135-0) contains supplementary material, which is available to authorized users.

## Background

Intellectual disability (ID) is one of the most common neurodevelopmental disorders (NDDs), affecting nearly 3% of the population worldwide. ID has a complex etiology resulting from the combination of environmental and genetic factors [1]. Relatively recent approaches to the identification of copy number variations (CNVs), have highlighted the relevance of rare de novo*,* and essentially private mutations that contribute to a significant proportion of the risk of NDDs, being presently an unavoidable element of diagnosis in the field of Neuropsychiatry, Neuropediatrics and Neurodevelopmental Pediatrics.

A substantial number of ID patients have CNVs resulting from deletions or duplications [2, 3]. The frequency of detection of chromosome abnormalities and/or genomic rearrangements in patients with NDDs by array comparative genomic hybridization (aCGH) depends mainly on the patient inclusion clinical criteria and on the microarray design; nevertheless, detection rates are usually higher in patients with ID/developmental delay (DD) that also present malformations or dysmorphic features and more severe cognitive impairment [2]. The characterization of these CNVs in different patient cohorts as well as in the general population is necessary to clarify their clinical relevance and establish adequate genotype-phenotype correlations [4].

We present the results obtained by studying 325 Portuguese patients with idiopathic ID using aCGH, in whom we found known and new candidate pathogenic CNVs. As expected, the great majority of the detected CNVs were rare and restricted to one patient/family; nevertheless, the efforts towards their characterization represent a step forward in order to clarify their clinical and molecular significance.

## Results

### Global data

From the 325 patients, 30.1% had at least one non-polymorphic CNV detected by aCGH (Part 1 of Additional file 1: Table S1): 8% had pathogenic CNVs, 5.2% had likely pathogenic CNVs and 16.9% had genomic variants of unknown significance (VOUS). The remaining 69.9% patients had only known polymorphic CNVs.

### Pathogenic CNVs

The pathogenic CNVs detected were mainly de novo CNVs, including deletions at 1p36.23-p36.21, 2p13.1–13.3, 3q22.1-q23, 5p15.33-p15.32, 6q25.3, 7q11.23, 8p23.1, 11q24.2-q25, 12q24.21-q24.22, 16p11.2, 17q21.31, 22q11.21 and 22q13.3, as well as duplications at 1q21.1, 12q24.21, 9q34.13–34.3, 13q12.12-q34, 14q32.31-q32.33, 14q32.33, 15q11.2-q13.1, 16p13.11, 21q11.2-q22.11, Xp11.22 and Xq28 (see Table 1 for the list of all patients and findings). For most of these CNVs there are reports in the literature describing the phenotypic and genetic findings for similar patients, therefore only some particular cases are described in detail and discussed in Part 1 of Additional file 1, namely: (a) the interstitial deletion at 1p36.23-p36.21 found de novo in patient R1, of interest since interstitial deletions in this region are rarely described in association with NDDs; (b) the deletion at 3q22.1-q23 found de novo in patient R3, which reinforces the association of deletions affecting *FOXL2* gene with blepharophimosis syndrome; (c) 7q11.23 deletions, detected in two non-related patients (C2 and R29), neither of whom presents the classical Williams-Beuren syndrome phenotype; (d) the 22q13.3 deletion found in patient C7, due to the incomplete overlap of the patient’s phenotype with that previously described for Phelan-McDermid syndrome; (e) the 9q34 duplications, detected in two non-related patients (C19 and R14): patient C19 has an intragenic *EHMT1* duplication and a clinical presentation that overlaps the core phenotype of Kleefstra syndrome, commonly caused by deletions or point mutations affecting the *EHMT1* gene; patient R14 has three de novo duplications at 9q34.13-q34.3 (affecting the whole *EHMT1* gene), at 14q32.31-q32.33 and at 14q32.33, illustrating the difficulty to ascertain the specific role of each imbalance in complex rearrangements. We also included in this category CNVs occurring in risk-associated *loci*.

**Table 1:** Clinical overview of RC patients for whom non-polymorphic CNVs vs likely benign and polymorphic CNVs were detected in the aCGH

Pathogenic + Likely pathogenic (*n* = 23)	Polymorphic CNVs (*n* = 134)
Gender	Gender
Males 15 (65%)	Males 84 (63%)
Females 8 (35%)	Females 50 (37%)
ID	ID
Syndromic 19 (83%)	Syndromic 74 (55%)
Non-syndromic 4 (17%)	Non-syndromic 60 (45%)
Borderline 1 (4%)	Borderline 8 (6%)
Mild 15 (65%)	Mild 75 (56%)
Moderate 6 (26%)	Moderate 30 (22%)
Severe 0 (0%)	Severe 15 (11%)
Profound 1 (4%)	Profound 6 (4%)
History	History
Sporadic 11 (48%)	Sporadic 54 (40%)
Family history of ID 15 (65%)	Family history of ID 80 (60%)
Co-morbidities	Co-morbidities
Congenital anomalies 11 (48%)	Congenital anomalies 64 (48%)
Epilepsy 2 (9%)	Epilepsy 19 (14%)
Microcephaly 4 (17%)	Microcephaly 23 (17%)
Macrocephaly 1 (4%)	Macrocephaly 13 (10%)

### Likely pathogenic CNVs

**Fig. 1:**
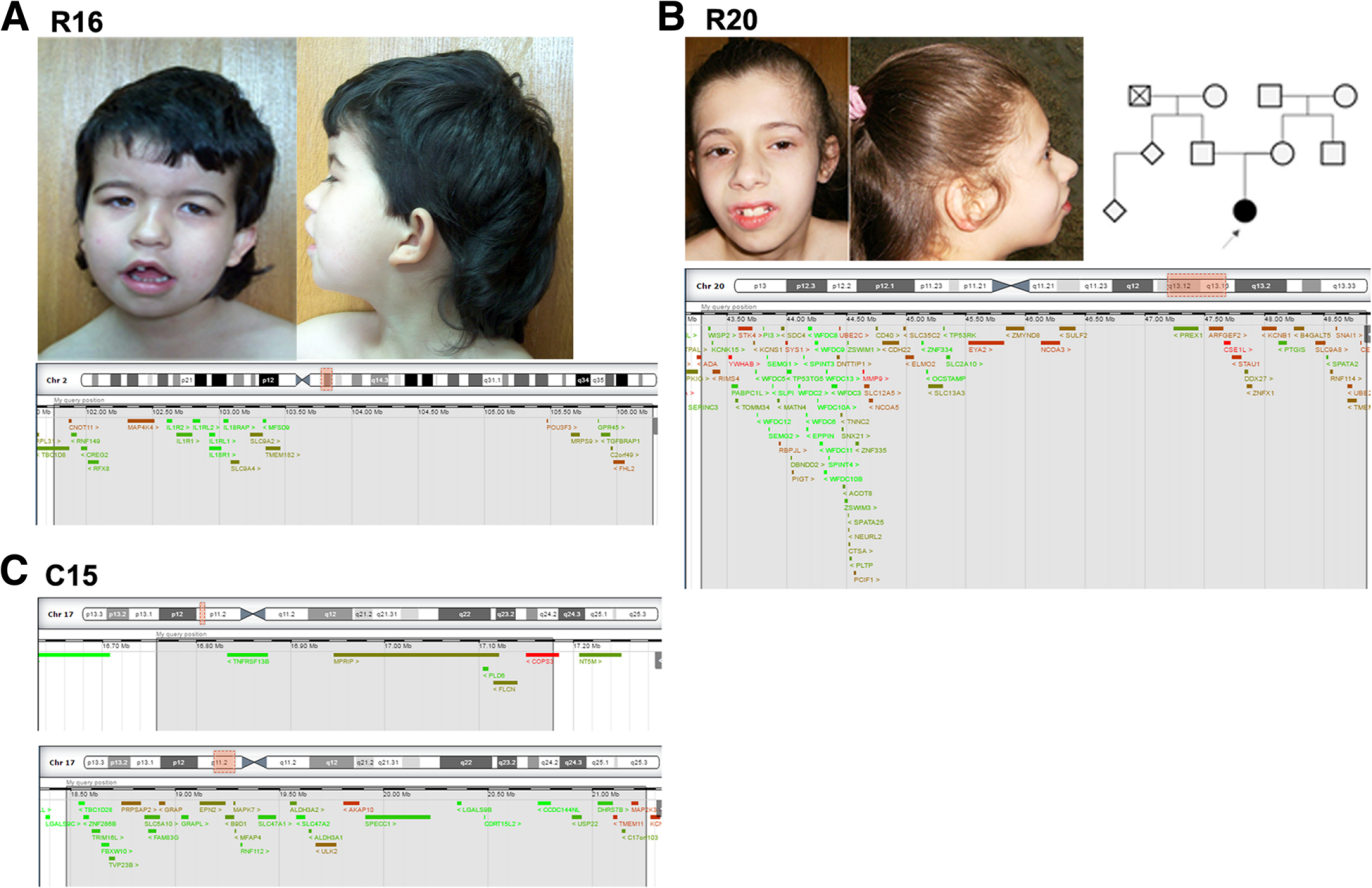
Facial appearance of patients and schematic representation of the deletions. **a** Patient R16 facies, with low set posteriorly rotated ears, anteverted ears with simplified helix, temporal narrowing with prominent metopic suture, arched eyebrows, synophrys, bilateral epicanthal folds, bulbous nasal tip, thin upper lip, open mouth with downturned corners, micrognathia; pedigree and deleted region of chromosome 2 (highlighted in red in the chromosome scheme (above) and in grey in the genes’ portion (below), adapted from DECIPHER). **b** Patient R20 facies, with wide forehead, strabismus, high nasal bridge, wide base of nose, bulbous nasal tip, short and smooth philtrum, thin upper lip with effaced cupid’s bow, prominent central incisors and micrognathia; pedigree and deleted region of chromosome 20. **c** Patient C15: deleted region in chromosome 17

**Fig. 2:**
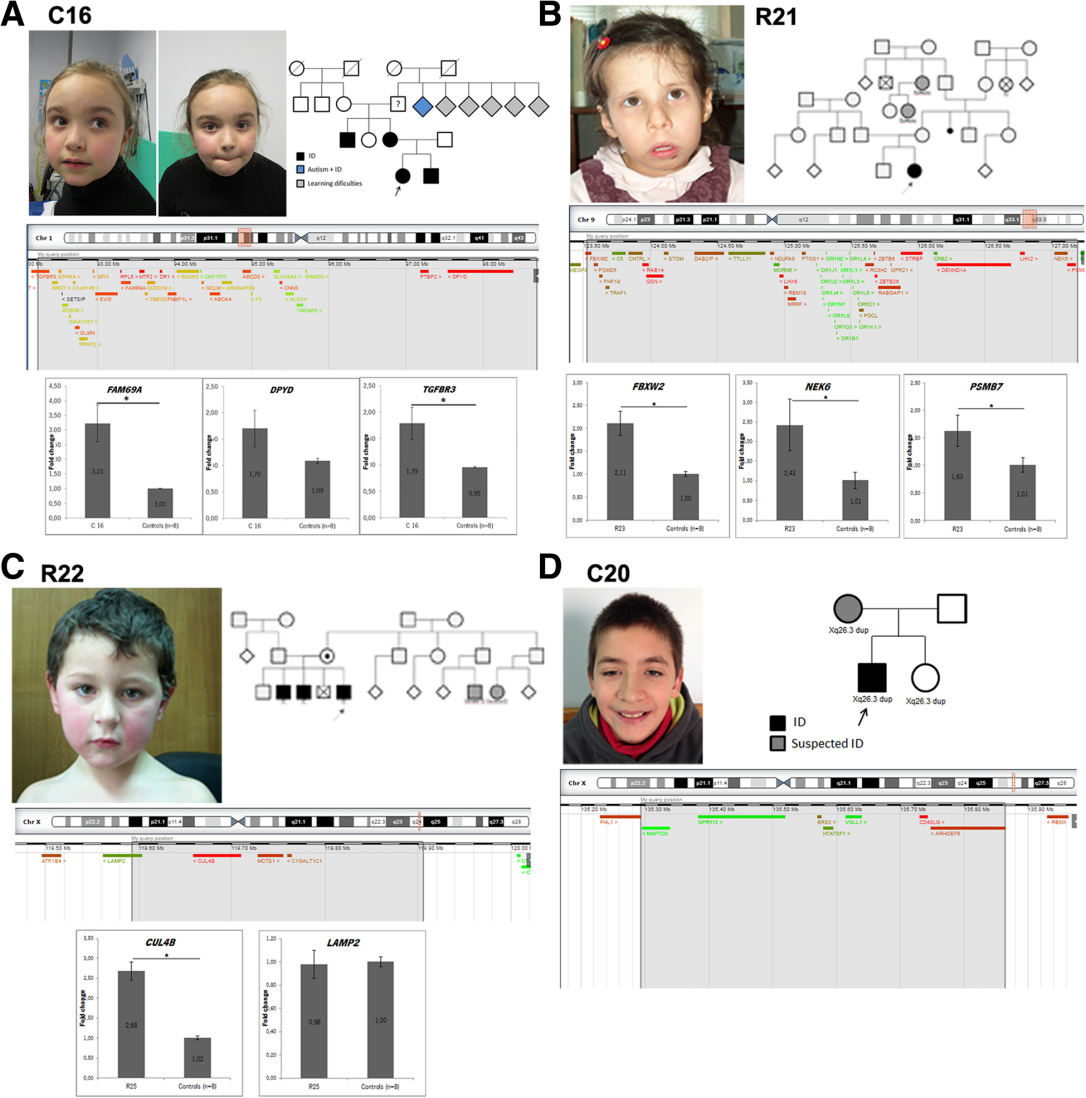
Overview of some patients with likely pathogenic duplications. **a** Patient C16 - facial appearance: mildly dysmorphic, with high forehead and frontal bossing, thick eyebrows and mildly anteverted nares; pedigree, schematic representation of the duplicated 1p region and expression pattern for genes *FAM69A*, *DPYD* and *TGFBR3*. **b** Patient R21 - facial appearance: large forehead, sparse lateral eyebrows, epicanthal folds, large nose, anteverted nares, long smooth philtrum, downturned corners of mouth and micrognathia; pedigree, schematic representation of the triplicated 9q region and expression pattern for genes *FBXW2*, *NEK6* and *PSMB7*. **c** Patient R22 - facial appearance: mildly dysmorphic with large forehead and frontal central hair whorl; pedigree, schematic representation of the duplicated Xq region and expression pattern for *CUL4B* and *LAMP2* genes. **d** Patient C20 - facial appearance: mildly dysmorphic patient with thick eyebrows, wide palpebral fissures and thin upper lip; pedigree and schematic representation of the duplicated Xq region. *B2M* and *PPIB* were used as housekeeping genes; * *p* < 0.05 (Student t-test)

Likely pathogenic CNVs were detected in 5.2% of patients in this study (Table 2; Figs. 1 and 2). They comprise candidate ID-causative *loci* located in 1q43-q44, 2q11.2-q12.2, 7q33, 10q26.3, 17p11.2 and 20q13.12-q13.13 (losses); 1p22.1-p21.3, 7q33, 9q33.2-q33.3, 9q34.3, Xq24 and Xq26.3 (gains) (Table 2). Patients with 1q43-q44, 7q33 and 10q26.3 CNVs have been described elsewhere in detail [5–7]; the patient with a 9q34.3 gain is described together with patient R14 in Part 1 of Additional file 1; therefore, we focus next on the remaining candidate *loci*.

**Table 2:** List of pathogenic CNVs

Patients	Gender	Alteration (Hg19)	Type	Size (Mb)	Genes	Key gene(s) involved	Associated syndrome	Phenotype overlap	Inheritance	Confirmation	Array platform	Ref
R1	Male	arr 1p36.23-p36.21(8,593,674-15,396,672)x1dn	del	6.7	86	*ANGPTL7, CASZ1, MAD2L2, RERE*	–	–	de novo	NP	1	–
R2	Male	arr 2p13.1-p13.3(70,894,906-74,986,518)x1dn^c^	del	4	62	*CYP26B1, EXOC6B*	–	–	de novo	NP	1	Wen J, 2013
R3	Male	arr 3q22.1-q23(131,415,639-141,618,552)x1dn	del	1.020	65	*FOXL2*	BPES	Yes (eye features)	de novo	NP	1	–
C1	Male	arr 5p15.33-p15.32(204,849-5,014,883)x1	del	4.81	30	*TERT [CTNND2 not involved]*	–	–	ND	NP	2	–
R4	Male	arr 6q25.3(156,012,754-158,804,494)x1dn^c^	del	2.6	14	*ARID1B*	Coffin-Siris syndrome	Yes	de novo	NP	1	Santen GW, 2013
C2	Male	arr 7q11.23(72,721,760-74,140,846)x1	del	1.419	28	*BAZ1B, STX1A, WBSCR22, ELN*	Williams-Beuren syndrome	Partially	ND	NP	2	–
R5	Female	arr 8p23.1(7,039,276-12,485,558)x1dn	del	5.5	70	SOX7, *GATA4*	8p23.1 deletion syndrome	Yes (cardiac)	de novo	NP	1	–
C3	Male	arr 11q24.2-q25(125,232,584-134,446,160)x1dn	del	9.214	54	*KIRREL3, ETS1, FLI1, KCNJ1, KCNJ5, RICS*	–	Partially	de novo	qPCR	2	–
R6	Female	arr 12q24.21-q24.22(115,505,500-117,441,683)x1dn^c^	del	0.2	10	*MED13L*	–	Yes	de novo	qPCR	1	Adegbola A, 2015
C4	Male	arr 16p11.2(29,674,336-30,198,123)x1dn	del	0.524	29	*KCTD13*	16p11.2 deletion syndrome	–	de novo	NP	2	–
C5	Male	arr 17q21.31(43,710,371-44,215,352)x1	del	0.505	8	*CRHR1, MAPT, STH, and part of the KIAA1267 (KANSL1)*	17q21.31 deletion syndrome (Koolen-De Vries syndrome)	–	ND	NP	3	–
C6	Male	arr 22q11.21(18,894,835-21,505,417)x1	del	2.611	59	*TBX1*	22q11 deletion syndrome	–	ND	NP	2	–
C7	Male	arr 22q13.3(49,513,903-51,178,264)x1	del	1.664	39	*SHANK3*	22q13.3 deletion syndrome (Phelan-McDermid syndrome)	Partially	ND	NP	2	–
C8	Male	arr 1q21.1q21.2(146,106,723-147,830,830)x3dn	dup	1.7	17	*HYDIN2, PRKAB2*	1q21.1 duplication syndrome ^e^	Partially	de novo	qPCR	4	–
R7	Male	arr 1q21.1(145,883,119-148,828,690)x3pat	dup	2.5	23	*HYDIN2, PRKAB2, GJA5*	1q21.1 duplication syndrome ^e^	Yes	paternal	NP	1	–
R8	Male	arr 12q24.21(116,408,736-116,704,303)x3dn^c^	dup	0.3	2	*MED13L*	–	Yes	de novo	qPCR	1	Adegbola A, 2015
C9	Male	arr 13q12.12-q34(23,749,431-115,083,342)x2.15^a^	dup	91.33	##	*–*	Trisomy 13 (mosaicism)	Yes	ND	Karyotype^d^	2	–
C10	Female	arr 15q11.2-q13.1(22880274–29,331,964)x3mat	dup	6.45	111	*CYF1P1, NIPA2, NIPA1, MKRN3, NDN, MAGEL2, SNURF/SNRPN, UBE3A GABRB3*	15q11-q13 duplication syndrome^b^	Yes	maternal	NP	2	–
C11	Female	arr 16p13.11(15,034,010-16,199,882)x3	dup	1.166	11	*NDE1*	16p13.11 duplication syndrome ^e^	–	ND	NP	5	–
R9	Male	arr 16p13.11(15,421,671-16,443,968)x3mat	dup	1	19	*NDE1*	16p13.11 duplication syndrome ^e^	Yes	maternal	NP	1	–
R10	Male	arr 16p13.11(15,484,180-16,308,344)x3mat	dup	0.8	9	*NDE1*	16p13.11 duplication syndrome ^e^	Yes	maternal	NP	1	–
C12	Male	arr 21q11.2-q22.11(14,417,523-34,894,625)x3	dup	20.47	110	*DSCR1, DSCR2, DSCR3, DSCR4, APP*	–	No	ND	NP	2	–
R11	Male	arr Xp11.22(53,569,653-53,769,748)x2mat	dup	0.2	3	*HUWE1*	–	Yes	maternal	qPCR	1	–
R12	Male	arr Xq28(152,348,378-155,228,013)x2dn	dup	2.8	78	*MECP2*	MECP2 duplication syndrome	Yes	de novo	NP	1	–
R13	Male	arr Xq28(153,130,545-153,602,293)x2mat	dup	0.5	16	*MECP2*	MECP2 duplication syndrome	Yes	maternal	NP	1	–
R14	Male	arr 9q34.13-q34.3(135,767,911-141,153,431)x3dn	dup	5.516	135	*EHMT1, RXRA, GRIN1, UAP1L1*	9q34 duplication syndrome	Partially	de novo	NP	1	–
		arr 14q32.31-q32.33(102,959,110-104,578,612)x3dn	dup	1.620	22	*MARK3, KLC1, EIF5*	–	–	de novo	NP	1	–
		arr 14q32.33(105,104,831-106,531,339)x3dn	dup	1.427	24		–	–	de novo	NP	1	–

Patients R1 to R14: from research cohort; Patients C1 to C12: from clinical cohort; *NP* Not performed, *ND* Not determined; (^a^): mosaicism; (^b^) methylation status for *SNRPN* is normal (studied by MLPA); (^c^): Published in detail elsewhere; (^d^): karyotype revealed a balanced translocation between chromosomes 13 and 14, resulting in mosaic trisomy 13; (^e^): Other causes of disease were not excluded therefore the variant might not explain the total phenotypic presentation. Array platform 1: Agilent 180 K; 2: KaryoArray®v3.0 (Agilent 8x60k); 3: Affymetrix CytoScan HD array; 4: Affymetrix CytoScan 750 K; 5: Agilent Whole Genome 244 K

#### 2q11.2-q12.2 deletion

Patient R16 is a 17 year old girl with syndromic ID, cerebral ventricular enlargement, dysmorphic features and hirsutism. She carries a de novo 4.5 Mb deletion at 2q11.2-q12.2 affecting 26 genes, of which *MAP4K4, FHL2, POU3F3* and *CNOT11* have the highest haploinsufficiency score in DECIPHERr [8]. *POU Class 3 Homeobox 3* (*POU3F3*) was previously reported deleted in a boy with ID and dysmorphic features (such as flat nose, prominent ears, large eyebrows and low hairline) [9], similar to those of our patient. This gene encodes a transcription factor present in post-mitotic cells and plays a role in neurogenesis and the correct destination of migratory neurons in the cerebral cortex in the mouse [10], thus standing out as a good candidate for the DD/ID in the patient.

#### 17p11.2 deletions

Patient C15 is a 10 year old boy referred for consultation for DD, namely language and motor impairment, ataxia and some dysmorphic features, including hypertelorism, strabismus and low-set ears. It was not possible to reevaluate for IQ testing, but at the time of first evaluation he had no formal cognitive deficit (according with the GMDS score when he was 5 years old) and cerebral magnetic resonance imaging (MRI) showed no alterations. He has what appear to be two consecutive deletions at 17p11.2: a 420.6Kb deletion, that encompasses 5 genes, and a 2.77 Mb deletion that encompasses 36 genes. He has inherited them from his mother, who has confirmed learning difficulties, although she has completed the 6th grade. These deletions partially overlap the region involved in Smith-Magenis syndrome (SMS); however, the phenotype of the patient and mother is not similar to that of SMS, and the deletion does not affect the retinoic acid induced 1 (*RAI1*) gene, thought to cause most of the SMS core phenotype [11]. Among the genes affected by patient C15’s deletions, there are several others whose function could potentially contribute for his phenotype (detailed in Part 1 of Additional file 1).

#### 20q13.12-q13.13 deletions

Patient R20 is a 16 year old girl with mild ID (IQ = 56), speech delay, MIC and facial dysmorphisms. Brain imaging studies revealed no structural alterations. She also has astigmatism and attention deficit hyperactivity disorder (ADHD). She carries a de novo 5.5 Mb deletion at 20q13.12-q13.13 encompassing 123 genes. Among these, the genes *KCNB1, PIGT, CTSA, SLC2A10* and *ARFGEF2* were associated with human disease (detailed in Part 1 of Additional file 1).

#### 1p22.1p21.3 duplications

Patient C16 is a 7 year old girl with motor and speech delay, with a global DQ of 56.3 (GMDS). She carries a maternal 1p22.1p21.3 duplication of 6.461 Mb that affects 44 genes. Her mother has completed the 6th grade although with 2 in-grade retentions and always showing learning difficulties, especially in language skills. The girl has a 10 year old brother suspected of having cognitive deficit: he was not evaluated yet, but he is attending the 2nd grade and does not yet know how to read. There is also a positive history of learning difficulties on the maternal grandfather’s family side. The duplication affects several genes (Fig. 2a), including the *FAM69A* gene, which encodes a member of the FAM69 family of cysteine-rich type II transmembrane proteins. FAM69 proteins are thought to play a fundamental role in the endoplasmic reticulum, in addition to specialized roles in the vertebrate nervous system, according to a brain-specific or brain-including expression pattern [12]. Consistently, several *FAM69* genes have been linked to neuropsychiatric disorders: *C3ORF58* (*DIA1*) with autism [13]; *CXORF36* (*DIA1R*) with X-linked ID [14] and *FAM69A* with schizophrenia and bipolar disease [15]. Even though the contribution of the excess of dosage for NDDS is still unknown, this gene can be considered a good candidate to explain the disease in the patient.

#### 9q33.2-q33.3 triplication

Patient R21 is a 17 year old girl with mild ID (IQ = 53) and familial history of ID. During the neonatal period she presented seizures (flexion spasms and later generalized tonic-clonic), controlled with Phenobarbital, which was discontinued at 23 months; EEG initially showed lateral paroxystic activity, bilaterally, and a normal result at 6 months; brain MRI was normal. Additionally, she presented dysmorphic facial features (Fig. 2), a muscular ventricular septal defect that closed spontaneously, hypothyroidism, hypotonia, global DD, growth deceleration (height and weight around the 3rd centile after 12 months) with normal head size, around the 75th centile, delayed bone maturation (~ 3 years), growth hormone deficiency and short neck. She carries a 3.6 Mb de novo triplication at 9q33.2-q33.3 that affects 60 genes. Of those, only the *CRB2* gene is associated with a human disease. Moreover, this triplication apparently disrupts the *FBXW2* gene that encodes for an F-box protein. F-box proteins are one of the four subunits of ubiquitin protein ligases, called SCFs. SCF ligases bring ubiquitin conjugating enzymes to substrates that are specifically recruited by the different F-box proteins. Components of this complex, such as *CUL4B,* have been involved in ID pathogenesis [16]. Also included in the CNV are the *LHX2* and *LHX6* genes, both encoding transcription factors described to play roles in brain development [17, 18]. Additionally, *LHX2* was also described to be involved in osteoclast differentiation and its overexpression inhibits skeletal muscle differentiation [19]. *LHX6* is also known to play a role in cranial and tooth development [20], hence these genes could be of relevance to the cranioskeletal phenotype of the patient.

Based on the location within the triplication region and the expression levels described we selected the *FBXW2*, *NEK6* and *PSMB7* genes (detailed in Part 1 of Additional file 1) to study at the mRNA level in peripheral blood in the patient. The three genes had an increased expression when compared to controls (Fig. 2b). For *NEK6* these findings are in accordance with the fact it is included inside the triplicated region. Regarding *FBXW2* and *PSMB7,* we had hypothesized that their expression could be diminished since they are located at the breakpoints, which we concluded not to be the case. To the best of our knowledge no mutations in any of these three genes were reported in human NDDs, making their involvement in our patient’s symptomatology difficult to confirm at this stage.

#### Xq24 duplication

Patient R22 is a 14 year old boy with borderline IQ (IQ = 80) and a familial history of ID (two brothers and cousins with ID), an apparently benign cardiac arrhythmia, overweight (BMI 23.6 Kg/m^2^ P90), stereotypies and ADHD. He carries a 0.3 Mb maternally inherited duplication at Xq24 affecting four genes (*CUL4B, LAMP2, C1GALT1C1, MCTS1*), his mother being asymptomatic. Both point mutations and large deletions in the *CUL4B* gene are described as causative of X-linked ID and cerebral malformations [21, 22]. *CUL4B* is a scaffold protein member of the cullin family that works in the formation of protein complex that acts as an E3 ubiquitin ligase catalyzing the polyubiquitination of protein substrates. *CUL4B* was found to be responsible for *TSC2* degradation in neocortical neurons positively regulating mTOR activity in those cells [23]. Additionally, *CUL4B* also targets *WDR5* for ubiquitylation leading to its degradation in neurons nucleus, which causes impaired neurite outgrowth [24]. However, to our knowledge, there is only one 47.2 Mb duplication encompassing *CUL4B* (and other genes) described in a patient with ID [25], the present case being the first small, non-disruptive *CUL4B* duplication described in a patient with ID. *CUL4B* is entirely duplicated in the patient and its expression in peripheral blood cells is increased, leading to us to believe that the disorder in the patient is in fact driven by a dosage increase in *CUL4B*. The *LAMP2* gene, located in the duplication breakpoint and encoding a protein with roles in autophagy/lysosomal function, does not present altered expression in the patient, suggesting that it may not be contributing to this phenotype (Fig. 2c).

#### Xq26.3 duplication

Patient C20 is a 17 year old boy referred to the consultation due to general DD. He carries a 570.1Kb duplication at Xq26.3 inherited from his mother, who has a suspicion of some cognitive impairment but for whom no formal intellectual assessment was possible. He has a global DQ of 57.1 (evaluated at the age of 10 years), scoring below the average in all GMDS sub-scales, namely on language and eye-hand co-ordination, and is described as a friendly boy. He has speech delay, dolichocephaly and several dysmorphisms, including micrognatia, syndactyly and clinodactyly. His younger sister (8 years old) also carries the duplication but has no ID and has a normal development for her age which, this being an X-linked gene, is not incompatible with the causality of disease. The duplication encompasses the several genes (Fig. 2d) including the *ARHGEF6* gene. *ARHGEF6* encodes for a protein that belongs to a family of cytoplasmic proteins which activate the Rho proteins by exchanging bound GDP for GTP. These Rho GTPases play a fundamental role in numerous cellular processes linked to the organization of the cytoskeleton, cell shape, and motility [26]. *ARHGEF6* specifically has been implicated in the regulation of spine morphogenesis and loss of function (LoF) mutations have been found in patients with X-linked ID [27]. A 2.8 Mb duplication in Xq26.2-Xq26.3 has also been described in two brothers with ID and the *ARHGEF6*, *PHF6*, *HPRT1* and *SLC9A6* genes have been identified as potential contributors to those patients’ phenotype [28]. When compared to this publication, we can see that our patient’s duplication is smaller and affects only the *ARHGEF6* gene; nevertheless, the phenotypic similarities between our patient and those described by Madrigal and colleagues (namely ID, dolichocephaly and facial dysmorphisms) suggest a determinant role for *ARHGEF6* gene in phenotypes associated with Xq26 microduplications [28]. Expression data in the periphery for some of the genes involved in the duplication didn’t retrieve results that we could interpret.

### CNVs of unknown significance

In the VOUS group, we included CNVs which did not encompass a known NDDs-related CNV region and for which (i) pathogenicity was not sufficiently supported by biological data, and/or (ii) similar copy number changes were described in control databases, and/or (iii) were inherited from a parent for whom the clinical presentation was not known. For 50% of these cases, inheritance from parents was not possible to determine due to parental sample unavailability, thus reducing our ability to interpret their clinical significance. A summary of the VOUS identified in this study is presented in Part 1 of Additional file 1: Table S2).

## Discussion

This study of a cohort of ID patients in whom most common causes of disease had been excluded allowed us to find a reliable cause of disease in 8% of patients and to propose novel candidate ID *loci* in 5.2%. Making a stricter analysis and considering only the variants associated (or likely associated) with disease we can consider that this yield is comparable with several other similar studies, in which percentages ranging between 8.5 and 16% were achieved [29–31]. The CNVs classified as pathogenic often appear de novo and affect (in general) dozens of genes. Some difficulties arose when classifying several of these CNVs as, in some cases, although they occurred in known syndrome regions not all the patients carrying them presented the major clinical features established for that particular syndrome. In fact, even these well-established pathogenic CNVs can be associated with a broad and distinctive phenotypic presentation, as observed in patients C2 and R29, both with WBS associated deletions but not presenting the full-blown phenotype of this syndrome. In this perspective, we believe that the main contributions of this work are: (I) the reporting of new patients with CNVs in regions associated with identified syndromes but with different clinical presentations; (II) the reporting of novel candidate ID-causative *loci* at 2q11.2-q12.2 (del), 7q33 (del and dup), 10q26.3 (del), 17p11.2 (del), 20q13.12-q13.13 (del), 1p22.1-p21.3 (dup), 9q33.2-q33.3 (tri), 9q34.3 (dup), Xq24 (dup) and Xq26.3 (dup); (III) the study in patients with copy number gains of the mRNA expression in peripheral blood for genes located either inside the duplicated/triplicated regions and/or at the breakpoints, making it possible to determine if there is an actual effect of gene dosage at the transcription level. Many of the CNVs here detected by aCGH were rare and restricted to one patient/family, which made their contribution to the patient’s phenotype difficult to assess. Several of these have been therefore classified as VOUS and their clinical significance needs to be carefully addressed in future studies. Individually rare intermediate-size CNVs (frequency, ≤0.05%; ≥250 kb), and not necessarily assigned a priori as pathogenic, appear to be collectively common in unselected populations (10.5%), and have been associated with ID and negatively with educational attainment [4]; being so, even these should not be excluded as cause of disease but rather re-assessed in the face of accumulating information, in order to establish useful genotype-phenotype correlations. Nevertheless, one cannot exclude the possibility that some of these CNVs are unrelated to pathogenesis, namely in patients where no other genomic testing (such as whole-exome or whole-genome sequencing) was performed to rule out other causes, this being a potential limitation of this work.

### NDDs associated pathways: old and new genes

The likely pathogenic CNVs here proposed as novel candidate *loci* for ID encompass several genes that either were already associated with NDDs (like *CUL4B*) or are now proposed to have a role in ID and which can be grouped according to their function in several cellular aspects:

#### Transcriptional factors/cell cycle regulators/DNA repair proteins

Transcriptional regulation is an essential component of the neuronal differentiation programs and of the response to stimulation patterns underlying neuronal plasticity; genes involved in these pathways have been implicated in well-known NDDs, as is the case of *FOXL2* [32], *BAZ1B* [33], and *EBF3* [7]*.* This work revealed genes that appear to be good candidate *loci* for ID; of those, *POU3F3*, already described deleted in a patient with ID [9], stands as a strong candidate.

#### Chromatin modifiers/chromatin remodeling proteins

An excess of mutation genes encoding proteins involved in chromatin regulation have been described in NDDs [34]. *EHMT1* and *ARID1B* belong to this category and are known to be associated with ID for many years. Here we describe two more patients with duplications affecting the *EHMT1* gene, in one of which it was possible to show *EHMT1* overexpression. *ARID5A* encodes for a protein belonging to the ARID family of proteins with important roles in development, tissue-specific gene expression and proliferation control [35].

#### Ubiquitin signaling

Ubiquitin-mediated degradation of proteins is a crucial mechanism for cell maintenance and viability [36]. Several genes belonging to this pathway are described to be associated with NDDs, as is the case of *CUL4B* [21], shown here to be duplicated in two patients. *UBE2C* encodes a key component of the ubiquitin proteasome system (UPS) that participates in cell cycle progression and checkpoint control [37]. The *NEURL3* and *CNOT4* genes also encode for proteins with E3 ubiquitin-protein ligase activity; as for *FBXW2*, it encodes for one of the four types of subunits of SCF ubiquitin-protein ligases. Neither of these genes has been linked, until now, with NDDs, but our findings reinforce the idea that genes encoding for proteins belonging to the UPS are possible new candidate genes for NDD phenotypes.

#### Cytoskeleton regulation and organization, cell shape and motility

Several NDDs are caused by mutations in genes regulating neuronal migration, which often encode for proteins involved in the function of the cytoskeleton [38]. *TSC1,* involved in microtubule-mediated protein transport due to unregulated mTOR signaling [39], and *ARHGEF6*, here described in different CNVs, have been previously associated with NDDs [39, 40]. *B9D1* has been confirmed as a novel Meckel syndrome gene [41].

#### Intracellular vesicular trafficking and exocytosis

In this work we report a patient with a deletion encompassing *ARFGEF2,* previously described associated with epilepsy and ID (in the case of homozygous mutations) [42, 43]. The collection of patients presented herein also allowed the first description of *EXOC6B* gene haploinsufficiency in association with DD/ID (reported in detail in a dedicated publication) [44].

#### Signaling mediators/transducers/ receptor activity/transmembrane proteins

Disruption of synaptogenesis has been associated with ID and NDDs [45] and in this work we could identify CNVs in several genes associated with this pathway. *SEMA4C* gene encodes a transmembrane semaphorin which regulates axonal guidance in the developing nervous system [46]. Syntaxins, such as Syntaxin 1A, encoded by *STX1A* gene, are key molecules implicated in the docking of synaptic vesicles with the presynaptic plasma membrane [47]. Signaling processes are essential for proper cellular function and usually implicate enzymes, transmembrane proteins and voltage ion-channels whose disruption may be associated with disease [48]. Many of the genes described herein, including *CACNA1C, GPR45, TNFRSF13B, FAM69A, AKT3* and *CSE1L,* are associated with these pathways, highlighting once again the crucial contribution of proper cellular signaling and synapse development and function for ID/DD.

Of notice, and although our attempts of establishing genotype-phenotype correlations was mostly focused on dosage impact of individual genes (e.g. haploinsufficiency/overexpression), CNVs may also lead to disease through other mechanisms, namely gene fusion generation [49] and impact on genome architecture, for example Topological Associated Domain disruption, with impact on the expression of genes located outside the affected regions [50].

## Conclusion

The aCGH technology has for long been used in the research and clinical contexts allowing the delineation of many new microdeletion and microduplication syndromes. In the last decade a decrease in the rate at which new syndromes were described has been observed, most likely because the most frequent/recurrent CNVs were described in the early days of aCGH [51]. For the remaining and rarer (often “private”) forms, it is still important, however, to make an effort to share their clinical and genetic features as well as the CNV data, to support future diagnosis and establishment of genotype-phenotype correlations, as well as the identification of novel candidate genes for disease, as those advanced here.

## Subjects and methods

### Subjects

This work included the analysis of 325 ID patients (full IQ (FIQ) below 70 and borderline FIQ 70–80) of Portuguese origin (36.9% females, 63.1% males), of which 188 (mostly trios) were included in a research cohort (RC) and 137 were studied in the context of routine clinical genetics diagnostics (clinical cohort, CC), all being referenced as having NDDs (detailed description of inclusion criteria and clinical characterization provided in Part 1 of Additional file 1). For the RC we were able to obtain DNA for all the parents as well as a more extensive clinical description (see Table 3).

**Table 3:** List of likely pathogenic CNVs

Patients	Gender	Alteration (Hg19)	Type	Size (Kb)	Genes	Relevant genes involved	Confirmation	Inheritance	DGV controls	DECIPHER	Array platform	Ref
C13	Male	arr 1q43-q44(240,043,427-249,233,096)x1dn ^f^	del	3.7	18	*AKT3*	qPCR	de novo^d^	No	250,152, 250,915 (smaller)	1	Lopes F, et al., 2019
R15	Female	arr 1q43-q44(243,552,007-243,738,675)x1dn ^f^	del	0.19	2	*AKT3*	qPCR	de novo^d^	*No*	252,432 (smaller)	2	Lopes F, et al., 2019
C14	Male	arr 1q43q44(243,592,147-243,749,968)x1pat ^f^	del	0.16	2	*AKT3*	qPCR	paternal	*No*	252,432 (smaller)	1	Lopes F, et al., 2019
R16	Female	arr 2q11.2-q12.2(101,756,265-106,265,018)x1dn	del	4500	24	*MAP4K4, FHL2, POU3F3, CNOT11*	qPCR	de novo	No	251,756	2	–
R17, R18^e^	Male, Female	arr 7q33(133,176,651-135,252,871)x1mat ^f^	del	2076	23	*AGBL3, CNOT4, CALD1, EXOC4*	qPCR	maternal^a^	No	256,036	2	Lopes F, et al., 2018
R19	Female	arr 10q26.3(131,374,701-132,030,468)x1dn	del	600	3	*EBF3*	qPCR	de novo	3/6564^b^	No	2	Lopes F et al., 2017
C15	Male	arr 17p11.2(16,757,564-17,178,161)x1mat	del	420	5	*COPS3*	NP	maternal^a^	No	No	3	–
		arr 17p11.2(18,478,816-21,255,056)x1mat	del	2770	36	*EPN2, RNF112, ULK2, ALDH3A2, AKAP10, B9D1*	NP	maternal^a^	No	340,692 (smaller)		–
R20	Female	arr 20q13.12-q13.13(43,283,820-48,850,844)x1dn	del	5500	88	*KCNB1, PIGT, CTSA, SLC2A10, ARFGEF2*	NP	de novo	No	309	2	–
C16	Female	arr 1p22.1p21.3(92,227,986-98,689,243)x3mat	dup	6461	44	*FAM69A, TGFBR3, GLMN, EVI5, RPL5, MTF2, DR1, ABCA4, ABCD3, CNN3, PTBP2, DPYD*	qPCR	maternal^a^	No	318,358	1	–
C17,C18^e^	Male, Male	arr 7q33(134,598,205-134,815,177)x3mat ^f^	dup	216	2	*CALD1, AGBL3*	qPCR	maternal^a^	No	No	1	Lopes F, et al., 2018
R21	Female	arr 9q33.2-q33.3(123,525,064-127,187,619)x4dn	tri	3600	52	*CRB2, LHX2, LHX6, DENND1A, STRBP, RAB14, GSN, PSMB7, ZBTB26*	qPCR	de novo	No	No	2	–
C19	Female	arr 9q34.3(140540819–140,659,057)×3mat	dup	0.118	2	*EHMT1*	NP	maternal	1/2504 (smaller)	No	1	–
R22, R23^e^	Male, Male	arr Xq24(119,592,606-119,904,981)x2mat	dup^c^	300	4	*CUL4B, LAMP2, C1GALT1C1, MCTS1*	qPCR	maternal	No	No	2	–
C20	Male	arr Xq26.3(135,293,144-135,863,290)x2mat	dup	570	9	*ARHGEF6, CD40LG, BRS3, MAP7D3*	qPCR	maternal	No	No	3	–

Patients R15 to R23: from research cohort; Patients C13 to C20: from clinical cohort; *NP* Not performed; (^a^): inherited from an affected parent; (^b^): doubt regarding the quality of the call in these controls; (^c^) duplication may disrupt gene if located in tandem; (^d^) paternity and maternity confirmed; (^e^): siblings; (^f^): family described elsewhere. Array platform 1: Affymetrix Cystoscan 750 K; 2: Agilent 180 K; 3: KaryoArray®v3.0 (Agilent 8x60k)

### Methods

Genomic DNA was extracted from peripheral blood using either the Citogene® DNA isolation kit (Citomed, Portugal) manually or the QIAsymphony SP kit and apparatus. aCGH was performed using the following platforms Agilent 180 K (GPL15397); KaryoArray®v3.0 (Agilent 8x60k); Agilent Whole Genome 244 K (GPL10118); Affymetrix CytoScan HD (GPL1613) or CytoScan 750 K (GPL18637) (detailed description provided in Additional file 1).

### Data analysis

CNVs detected were classified using criteria adapted from those previously described elsewhere [3, 52] as: pathogenic, likely pathogenic, CNVs of unknown clinical significance (VOUS) (detailed description in Part 2 of Additional file 1). For simplification of terminology throughout the text and in the tables, the term CNV is used for pathogenic and likely pathogenic variants, as well as  VOUS. Polymorphic CNVs were not further considered in our analysis, except where specifically indicated (e.g. known risk *loci,* although relatively frequent, were considered pathogenic). All alteration are described in the tables as in the Decipher database (for example 12q24.21-q24). For CNV confirmation we performed qRT-PCR (7500-FAST Real Time PCR, Thermo Fisher Scientific, Waltham, MA, USA), using *SDC4* and *ZNF80* as reference genes (detailed description in Part 2 of Additional file 1; primers in Table S3). Total RNA was isolated from leukocytes using the QIAsymphony RNA Kit (QIAGEN GmbH, Germany), according to the manufacturer’s protocol. First-strand cDNA synthesized using SuperScript® III Reverse Transcriptase (RT) (Thermo Fisher Scientific, Waltham, MA, USA). Expression analysis was performed by quantitative real-time reverse transcription PCR (qRT-PCR) using Power SYBR Green® (Thermo Fisher Scientific, Waltham, MA, USA) (detailed description in Part 2 of Additional file 1; genes and primers listed in Table S4).

## Additional file

Additional file 1: **Figure S1.** Facial appearance of some patients carrying pathogenic variants. **Figure S2.** Clinical features of patients R14 and C19 and images of their CNVs. **Table S1.** Patients with altered aCGH results (i.e. with CNVs classified as non-polymorphic). **Table S2.** List of variants of unknown clinical significance (VOUS). **Table S3.** Primers used for quantitative PCR confirmation. **Table S4.** Primers used for expression studies. **Table S5.** OMIM entrance, haploinsufficiency score and constrain metrics for the selected genes in patient R16. **Table S6.** OMIM entrance, haploinsufficiency score and constrain metrics for the selected genes in patient C15. **Table S7.** OMIM entrance, haploinsufficiency score and constrain metrics for the selected genes in patient R20. **Table S8.** OMIM entrance, haploinsufficiency score and constrain metrics for the selected genes in patient C16. **Table S9.** OMIM entrance, haploinsufficiency score and constrain metrics for the selected genes in patient R21. **Table S10.** OMIM entrance, haploinsufficiency score and constrain metrics for the selected genes in patient C19. **Table S11.** OMIM entrance, haploinsufficiency score and constrain metrics for the selected genes in patients R22 and R23. **Table S12.** OMIM entrance, haploinsufficiency score and constrain metrics for the selected genes in patient C20. (DOC 11550 kb)

## Data Availability

All data generated or analysed during this study are included in this published article and in its supplementary information files.
