# Supplementary material for: Genomic imbalances defining novel intellectual disability associated loci
Source: Orphanet J Rare Dis. 2019 Jul 5;14:164. doi: 10.1186/s13023-019-1135-0 (PMC6612161; doi:10.1186/s13023-019-1135-0)
Supplement: Supplementary file 1 — Figure S1. Facial appearance of some patients carrying pathogenic variants. Figure S2. Clinical features of patients R14 and C19 and images of their CNVs. Table S1. Patients with altered aCGH results (i.e. with CNVs classified as non-polymorphic). Table S2. List of variants of unknown clinical significance (VOUS). Table S3. Primers used for quantitative PCR confirmation. Table S4. Primers used for expression studies. Table S5. OMIM entrance, haploinsufficiency score and constrain metrics for the selected genes in patient R16. Table S6. OMIM entrance, haploinsufficiency score and constrain metrics for the selected genes in patient C15. Table S7. OMIM entrance, haploinsufficiency score and constrain metrics for the selected genes in patient R20. Table S8. OMIM entrance, haploinsufficiency score and constrain metrics for the selected genes in patient C16. Table S9. OMIM entrance, haploinsufficiency score and constrain metrics for the selected genes in patient R21. Table S10. OMIM entrance, haploinsufficiency score and constrain metrics for the selected genes in patient C19. Table S11. OMIM entrance, haploinsufficiency score and constrain metrics for the selected genes in patients R22 and R23. Table S12. OMIM entrance, haploinsufficiency score and constrain metrics for the selected genes in patient C20. (DOC 11550 kb) [file 13023_2019_1135_MOESM1_ESM.doc]

**Additional file 1**

PART 1 – Detailed clinical and results description Page 3

Patients, inclusion criteria, and clinical characterization Page 3

Measures Page 4

Results Page 4

Pathogenic CNVs Page 4

1p36.23-p36.21 deletion Page 4

3q22.1-q23 deletions (BPES) Page 6

Supplementary figure 1 Page 7

7q11.23 deletions Page 7

22q13.3 deletions (Phelan-McDermid syndrome) Page 9

9q34, 14q32.31-q32.33 and 14q32.33 duplications Page 10

Supplementary figure 2 Page 12

Likely pathogenic CNVs Page 13

17p11.2 deletions Page 13

20q13.12-q13.13 deletions Page 14

9q33.2-q33.3 triplication Page 14

CNVs of unknown significance Page 15

Supplementary Table S1 Page 16

Supplementary Table S2 Page 17

PART 2 – Detailed methodology Page 22

aCGH Page 22

CNV classification criteria Page 22

Quantitative PCR confirmations Page 23

mRNA expression analysis Page 24

Methylation status Page 24

Supplementary Table S3 Page 25

Supplementary Table S4 Page 28

PART 3 - – Genes within likely pathogenic CNVs Page 30

Supplementary Table S5 Page 30

Supplementary Table S6 Page 30

Supplementary Table S7 Page 32

Supplementary Table S8 Page 33

Supplementary Table S9 Page 34

Supplementary Table S10 Page 35

Supplementary Table S11 Page 36

Supplementary Table S12 Page 37

Bibliography Page 38

**PART 1 – Detailed clinical and results description**

*Patients, inclusion criteria, and clinical characterization*

325 ID patients of Portuguese origin, of which 188 were included in a research cohort (RC) and 137 were studied in the context of clinical genetics diagnosis (clinical cohort, CC). For the RC group, the eligibility criteria used were stricter. In order to be included in the study the patient needed to have (I) documented DD/ ID (IQ test equal/below 70 for patients with more than 3 years or on basis of clinical evaluation by a pediatrician); (II) dysfunction/impairment in more than 2 areas of communication, self-care, home living, social/interpersonal skills, use of community resources, self-direction, functional academic skills, work, leisure and safety; (III) unknown aetiology, in spite of standard aetiological investigation; (IV) onset of ID during childhood; (V) previous normal investigations including biochemistry workup, high-resolution karyotype, Fragile X syndrome testing and FISH studies when clinically indicated, *ATRX* analysis, and pregnancy TORCH serologies if available. Patients with large genomic imbalances detectable by G-banded karyotyping, common environmental etiologies and common genetic etiologies were generally excluded. These patients were therefore considered to have idiopathic ID/DD. All the patients in this group were evaluated by a multidisciplinary team, which included a pediatrician and/or a neuropediatrician, a medical geneticist and a psychologist, and their information collected in a database in an anonymous manner. The database for clinical data collection was approved by the Portuguese national data protection committee (CNPD - Comissão Nacional de Proteção de Dados). In the CC, patients were enrolled from a private Genetics laboratory, and had been referenced as having ID, DD and/or congenital anomalies of unknown etiology. Written informed consent was obtained from the parents or persons in charge, for all the participants.

*Measures*

All participants were given an individually administered IQ test [Portuguese version of Wechsler Intelligence Scale for Children—Third Edition (WISC-III) (1); the participants’ primary caregiver had been administered the Vineland Adaptive Behavior Scale (VABS)-Survey form (2), using a semi-structured interview format. All measures were administered by experienced psychologists during routine clinical multidisciplinary assessments. The WISC-III evaluation instrument consists of thirteen subtests (M = 10; SD = 3) spread over two subscales: Verbal and Performance, each one evaluating a different aspect of intelligence (3). The performance of the subjects in the various subtests is clustered in three composite results: a general intelligence measure (FSIQ) and two ratios divided by the nature of its subtests: the VIQ, measurement of verbal intelligence, and the PIQ, a non-verbal intelligence measure (3).

***Results***

*Pathogenic CNVs*

For most of these CNVs there are reports in the literature describing the phenotypic and genetic findings for similar patients; therefore, only some particular cases will be discussed here.

*1p36.23-p36.21 deletion*

Patient R1 has a 6.7 Mb *de novo* deletion at 1p36.23-p36.21. This patient is an adult male (30 years old) with moderate ID (IQ= 49), microcephaly (MIC), broad nasal bridge, hypoplastic nares, microretrognathia, kyphosis, hypertelorism and telecanthus (Supplementary figure S1). Large terminal deletions in 1p36 are known to cause the 1p36 deletion syndrome (4), nevertheless, interstitial deletions in this region are quite rarely described associated with NDDs.

This deletion affects 126 genes among which four (*PEX14*, *PLOD1*, *NMNAT1* and *MTHFR*) had previously been associated with disease (5) and eight (*MTOR*, *ENO1*, *PIK3CD*, *RERE*, *NPPA*, *MAD2L2*, *MTHFR* and *KIF1B*) have a high haploinsufficiency score (6), possibly contributing for the phenotype. In the Decipher database (5), there are patients with similar overlapping deletions, of which three (266689, 248448 and 251601) also carry a *de novo* deletion and share similar clinical features, namely ID, MIC (patient 251601) and facial dysmorphisms.

*RERE* (Arginine-Glutamic Acid Dipeptide (RE) Repeats) encodes a protein that positively regulates retinoic acid signaling and that, when mutated in mice, leads to somite asymmetry (7). Also, in a mouse model with both a null and a hypomorphic Rere allele, several anomalies were described: microphtalmia, postnatal growth retardation, brain hypoplasia, decreased number of neurons in the hippocampus, cardiovascular malformations, hearing loss and renal agenesis (8). More recently, *de novo* heterozygous mutations in *RERE* were described in 10 patients with NDDs/ID, suggesting that *RERE* haploinsufficiency might contribute to ID-related phenotypes (9). Taking this into account, the haploinsufficiency of *RERE* may contribute (alone or in conjunction with the haploinsufficiency of other surrounding genes) for the growth retardation, short stature (height and weight), MIC and ID observed in the patient.

*MTOR* (Mechanistic Target Of Rapamycin (Serine/Threonine Kinase)) was described as mutated in patients with Smith-Kingsmore (SK) syndrome (AD), characterized by ID, macrocephaly, dysmorphisms and small thoraces (10). MTOR mutations were also described in a patient with epileptic encephalopathy without brain malformation (AD) (11). Besides ID, the patient didn’t present any major similarities with SK patients (apart from the alterations in head size which seem to be opposite between them). Nevertheless, *MTOR* is a key gene in a pathway in which several upstream and downstream components are known to play important roles in synaptic plasticity, and are associated with others NDDs (Fragile-X syndrome, Down syndrome, Tuberous sclerosis and autism) (12), hence it is a plausible contributor to the phenotype of this patient.

*3q22.1-q23 deletions (BPES)*

Patient R3 is a 15 year old boy with mild ID (IQ= 54), brachycephaly, cortical atrophy, cardiac interventricular communication, low weight and hypoplastic genitalia with hypospadias. He also presents a peculiar eye dysmorphism characterized by microphtalmia, epicanthus and ptosis (Supplementary figure S1). Interestingly, he carries a 10.2 Mb *de novo* deletion at 3q22.1-q23 affecting the *FOXL2* (Forkhead Box L2) gene, for which mutations are causative of blepharophimosis syndrome (BPES) (13). In recent years it has been shown that large deletions affecting *FOXL2* and its surroundings are often associated with ID combined with BPES, and this patient reinforces the association of deletions in this region with that phenotype.

**
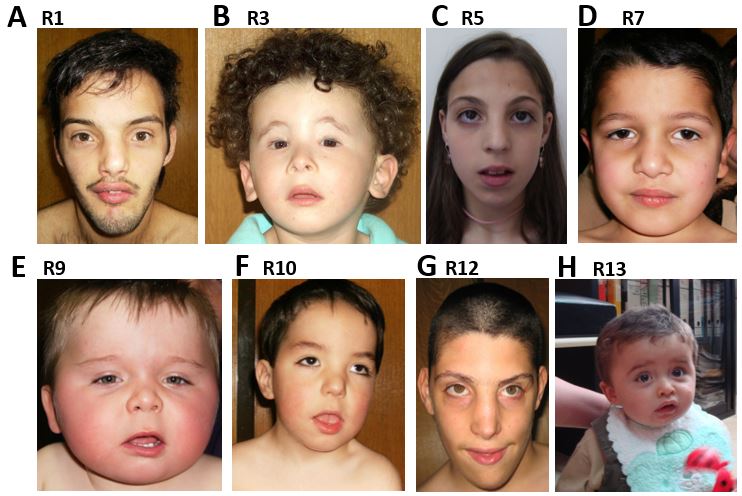
**

**Figure S1 – Facial appearance of some patients carrying pathogenic variants.** A, patient R1; B, patient R3; C, patient R5; D, patient R7; E, patient R9; F – patient R10; G, patient R12; H, patient R13.

*7q11.23 deletions*

Two non-related patients with 7q11.23 deletions were detected: C2 and R29.

Patient C2 is a 7 year old boy referred to the consultation due to DD, with previous normal investigations including high-resolution karyotype and Multiplex Ligation-dependent Probe Amplification (MLPA) analysis of subtelomeric regions. He was evaluated at 5 years and 7 months with Griffiths Mental Developmental Scales (GMDS) as having a global development quotient (DQ) of 58. In addition to ataxia, motor and language delay, he presents dysmorphic features that include flat nose, thin upper lip, narrow central incisors, large ears and hyperacusis, narrow girdles, mainly scapular, and large digits. An echocardiography did not reveal any anomaly; presently, he is underweight and does not present behavioral problems. He has a 1.419Mb deletion at 7q11.23 that overlaps the Williams-Beuren syndrome (WBS) critical region and encompasses 30 genes, including the *ELN* (elastin), *GTF2IRD1* (GTF2I repeat domain containing 1), *GTF2I* (general transcription factor II-I), *LIMK1* (LIM domain kinase 1) and *CLIP2* (CAP-Gly domain containing linker protein 2) genes, which, with exception of the *ELN* gene, associated with cardiac problems, were suggested to be linked to the specific cognitive profile and craniofacial features presented by WBS patients (14). Additionally, this patient also carries a 457.8Kb duplication at 15q13.3, that encompasses the *CHRNA7* and *OTUD7A* genes (~30% of the 15q13.3 microdeletion syndrome). It was not possible to test his parents to determine the origin of the CNVs.

Within the same region of chromosome 7, we found another deletion in patient R29, a 19 year old boy with severe ID (IQ=21) whose mother is suspected to have mild ID. He has cerebral atrophy, MIC, epilepsy and recurrent respiratory infections. Behavioral alterations include motor stereotypies, increased activity, sleep disturbances, aggressiveness and frequent verbal preservative repetitions (saying “hello” and “caress”). He carries a 0.4 Mb maternal deletion at 7q11.23 affecting 11 genes, including *BAZ1B*, *STX1A* and *WBSCR22* but not ELN. A neurocognitive assessment of the patient was performed as described previously for WBS patients (1,15), however, the patient was unable to understand most of the questions or requirement of several tasks, indicating a more severe ID than that expected in a classical WBS patient (16). In fact, he presents concurrent deficits in adaptive functioning, matching a profound ID classification (FSIQ – <20), with severe impairments in verbal comprehension, performance and working memory. This seems unlikely to be caused only by the 7q11.23 deletion.

*22q13.3 deletions (Phelan-McDermid syndrome)*

Patient C7 is an 8 year old boy who was referred to consultation at the age of 3, due to global DD, particularly in language and fine motor skills, but without behavioral alterations or dysmorphic features. He was thought to have neonatal hypotonia, as he was only able to sit alone at the age of 9 months. Presently, he has mild hypotonia, regular growth, does not have dysmorphic features and wears glasses, and is being followed in the strabismus/ophthalmological consultation. He has also cognitive deficits, scoring below the average in all GMDS sub-scales (locomotor, personal-social, language, eye and hand co-ordination, performance, practical reasoning). aCGH analysis showed that he has a 1.66Mb deletion at 22q13.3 that encompasses 39 genes including the SH3 and multiple ankyrin repeat domains 3 (*SHANK3*) gene, that encodes a multidomain scaffold protein (17) involved in synapsis (18). *SHANK3* is located within the minimum critical region of the Phelan-McDermid syndrome (PMS). Mutations in this gene are associated with several NDDs, including autism spectrum disorder (ASD), presented by more than 50% of the PMS patients (19), schizophrenia and bipolar disease, neurodevelopmental deficits (global DD, ID moderate to severe), absent or severely delayed speech, normal growth and several minor dysmorphic features (namely asymmetric face, maxillary prognathism, dysmorphic ears, ptosis and bulbous nose) (19). Patient C7 presents some clinical overlap with this presentation, namely the hypotonia, speech impairment and motor development delay, but no significant facial dysmorphisms. The severity of symptoms from PMS patients were recently linked to variability in the extent of mitochondrial dysfunction, caused by disturbance of several mitochondrial genes within the 22q13.3 critical region, like *SCO2*, *TYMP* and *CPT1B* all affected in this patient, as well as *NDUFA6*, *TRMU* and *ACO2* (20). This variability in the extent of the mitochondrial dysfunction could contribute for the incomplete overlap of patient’s phenotype with the one previously described.

*9q34, 14q32.31-q32.33 and 14q32.33 duplications*

9q34 duplications were detected in two unrelated patients: C19 and R14 (Supplementary figure S2). Patient C19 is a 12 year old girl with moderate ID, obesity (62.4Kg at 12 years, BMI=26.6 Kg/m^2^, P97), hypotonia and facial dysmorphisms, namely a coarse facies, midface hypoplasia (flat face appearance), synophrys, upslanting palpebral fissures, anteverted nares, downturned mouth and everted lower lip. She has a hoarse voice and speech articulation deficits. She presents behavioral problems, namely ADHD, medicated with methylphenidate, an obsessive-compulsive behavior towards food, and sleep disturbances (difficulties in falling asleep). Currently, her EEG analysis is normal, although she had one unprovoked seizure episode. She carries a maternal 118Kb 9q34.3-duplication from, at least, intron 2 to intron 11 of the *euchromatic histone-lysine N-methyltransferase 1* (*EHMT1*) gene. Haploinsufficiency for *EHMT1* causes Kleefstra syndrome (KS, MIM 610253), commonly through deletions or, more rarely, point mutations affecting this gene (21). Until now, only one case of KS caused by an intragenic *EHMT1* duplication was described (22), the duplication being identical to the one presented by this patient; additionally, patient C19´s clinical presentation overlaps the KS core phenotype (21). Although most cases of KS are caused by *de novo* mutations, two unrelated families were described in which affected children inherited a 9q34.3 deletion from a mildly affected mother who was somatically a mosaic for the deletion (23). Patient C19’s mother also carries the duplication, though not in mosaic state, and has confirmed psychiatric/cognitive problems.

Patient R14 is a 16 year old boy with mild ID (IQ= 57), facial dysmorphisms, hypochromic and *café-au-lait* spots. Behaviorally he presents stereotypies, obsessive and aggressive behavior and ADHD. He carries a rearrangement between chromosome 9 and chromosome 14 in which the 9q duplicated region is located in 14p. Moreover, the duplicated 14q32.31-q32.33 and 14q32.33 regions are likely located in tandem and may lead to the disruption of the genes involved in the breakpoints. It was not possible to determine in which chromosome 14 the 14q duplication is located (in the derivative or in the normal one) (Supplementary figure S2). This resulted in three *de novo* duplications: a 5.5Mb duplication at 9q34.13-q34.3, a 1.6 Mb duplication at 14q32.31-q32.33 and a 1.4 Mb duplication at 14q32.33. All of these might possibly contribute to his phenotype, making it difficult to ascertain the specific role of each imbalance.

Duplications affecting the 5’ region of the *EHMT1* gene and duplications or triplications encompassing the entire *EHMT1* gene have been observed in patients with neurodevelopmental impairment, speech delay, and ASD, suggesting that increased *EHMT1* dosage is associated with a neurodevelopmental phenotype (24). Interestingly, the expression of *EHMT1* and *TCS1* (for the 9q dup) was found to be increased in the peripheral blood of patient R14 when compared to controls. Since FISH studies have shown that the 9q34 duplicated region is not located in tandem, but in *trans* (Supplementary figure S2-C), most likely it will not originate any structural effect that could influence the expression of the genes within the region. As for the 14q32 duplicated region: I) the *INF2* mRNA expression was increased in the patient, in agreement with the fact that the entire gene is located inside the duplicated region; II) *TECPR2* expression was not altered. The portion of the transcript where the primers for *TECPR2* were designed is located outside the duplicated region. Nevertheless, we believe that if the duplicated region affected the expression of the gene, this would be still possible to observe since one of the alleles (the one located in the duplicated chromosome) would result in the degradation of the entire mRNA molecule (Supplementary figure S2).


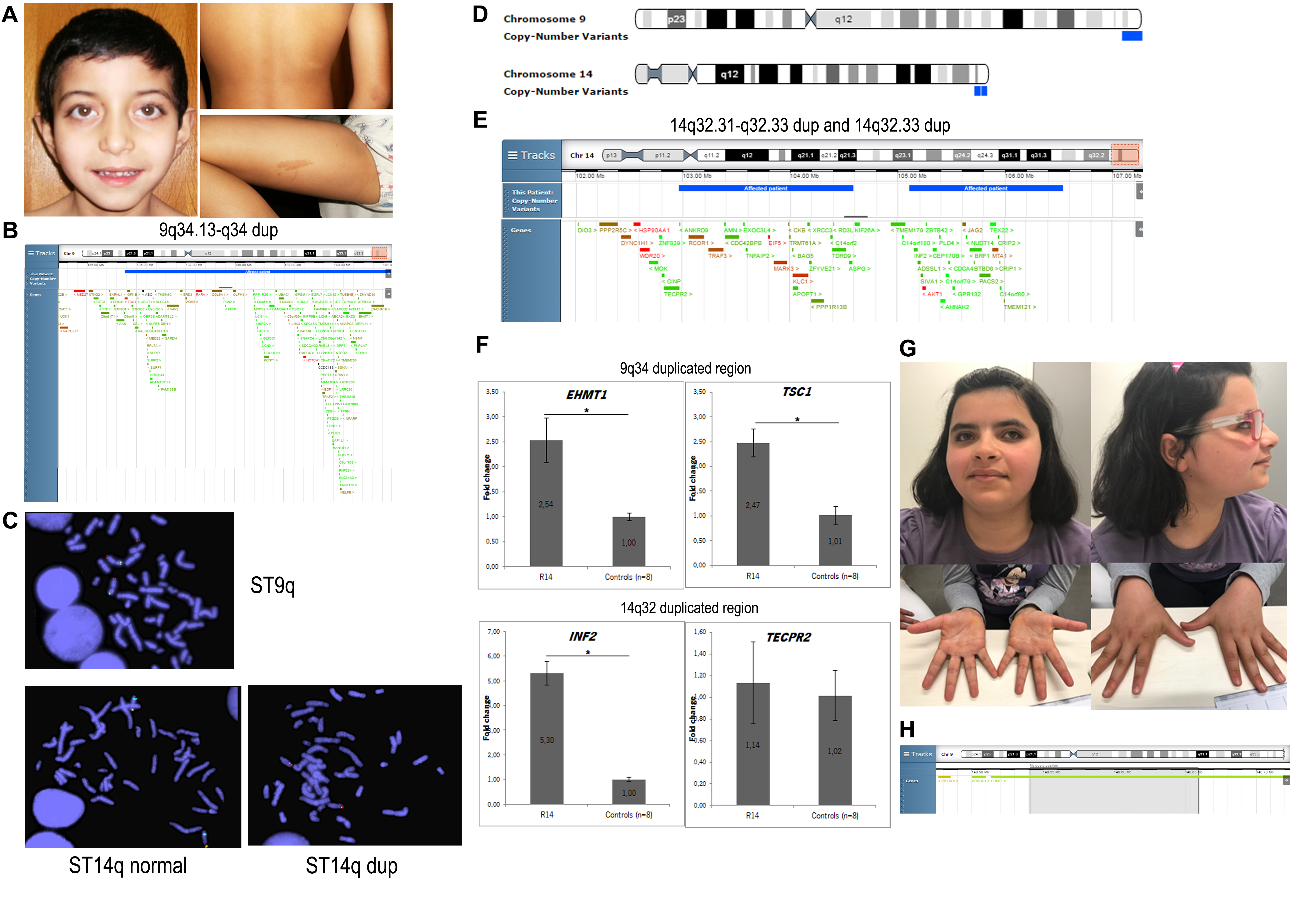


**Figure S2 – Clinical features of patients R14 and C19 and images of their CNVs.** (A) appearance of patient R14 (note the hyperpigmented spots); (B) schematic representation of the 9q31.13-q34 duplicated region in patient R14 (the blue bar indicates the duplicated region, adapted from DECIPHER); (C) FISH analysis for patient R14 (9q31.13-q34 – D9S325 - duplication in the top panel; 14q32 – D14S1420 - duplications in the lower panel) revealed the presence of a derivative chromosome in which the duplicated 9q region is located in the 14p. It was not possible to determine if the duplicated 9q portion is located in the duplicated or normal chromosome 14. The duplicated 14q region must be located in tandem. (D) Schematic representation of the duplicated regions in patient R14. (E) Scheme of both duplication in chromosome 14. (F) Expression pattern of *EHMT1*, *TSC1*, *TECPR2* and *INF2* genes. (G) Facial appearance of patient C19. (H) Duplicated 9q region in patient C19. (B2M was used as housekeeping gene; student t-test; p<0.05*).

*Likely pathogenic CNVs*

In this category were included only CNVs whose speculation of pathogenicity is supported in the literature, such as large CNVs that comprise good candidates for disease association. We provide here additional information concerning some CNVs detected.

*17p11.2 deletions*

There are other genes that could contribute for the phenotype of patient C15.

*EPN2* encodes for epsin2, a protein thought to be involved in clathrin-mediated endocytosis, found in a brain-derived clathrin-coated vesicle fraction (25). *RNF112* (ring finger protein 112, also known as *ZNF179*), which encodes a member of the RING finger protein family of transcription factors and is primarily expressed in brain (26) and *ULK2* (unc-51 like autophagy activating kinase 2), which encodes a protein similar to a serine/threonine kinase, the ortholog of which is known to be involved in axonal elongation in *Caenorhabditis elegans* (*C. elegans*) (27). Moreover, there are 3 OMIM genes affected by these deletions: *B9D1*, which encodes a B9 domain-containing protein, associated both with Meckel (28) and Joubert syndromes (both AR) (29); *ALDH3A2*, which encodes for a fatty aldehyde dehydrogenase that causes Sjögren-Larsson syndrome (AR) (30) when mutated and *AKAP10*, which encodes the mitochondrial A-kinase (PRKA) anchor protein 10, which may be associated with increased risk of arrhythmias and sudden cardiac death (31). All these being recessive disorders, the presence of these deletions in heterozygosity is unlikely to cause the phenotype. Nevertheless, a phenotype modifying effect cannot be excluded.

*20q13.12-q13.13 deletions*

Patient R20 carries a deletion that encompasses several genes, among which the genes *KCNB1, PIGT, CTSA, SLC2A10* and *ARFGEF2* were associated with human disease, whereas *MMP9, CSE1L* and *YWHAB* have very high haploinsufficiency scores in Decipher. *KCNB1* (Potassium Channel, Voltage Gated Shab Related Subfamily B, Member 1) encodes a potassium channel in which *de novo* mutations were found in patients with epilepsy and NDDs (32). The *ADP ribosylation factor guanine nucleotide exchange factor 2* (*ARFGEF2*) gene encodes a protein involved in the activation of ADP-ribosylation factors (ARFs) and is required for vesicle and membrane trafficking in the Golgi (33). Homozygous mutations in this gene have been described in patients with epilepsy, periventricular heterotopia with MIC, movement disorders and ID (34,35). Even though the clinical presentation is not completely overlapping (epilepsy not being a feature of disease in this case), the previous description of *KCNB1* and *ARFGEF2* genes with NDD make them good candidates for explaining the phenotype in the patient. However, the contribution of other genes within the deleted region cannot be excluded.

*9q33.2-q33.3 triplication*

Patient R21 carries a 3.6 Mb *de novo* triplication at 9q33.2-q33.3 that affects 60 genes. *NEK*6 and *PSMB7* were also selected for mRNA expression studies. *NEK6* encodes a protein kinase required for efficient mitotic spindle assembly (36) and found to be a key player in aging and cancer (37) *PSMB7* encodes a proteasome subunit thought to play a role in autophagy inhibition in cardiomyocytes (38). Besides these, two other genes also draw our attention: *DENND1A* (*DENN/MADD Domain Containing 1A*) is a guanine nucleotide exchange factor (GEFs) for the early endosomal small GTPase RAB35, that works on the endocytic branch of the synaptic vesicle cycle, and its ablation in hippocampal neurons leads to defects impaired synaptic vesicle endocytosis (39); *RAB14* (*RAS associated protein RAB14*) encodes a protein that works in the trafficking between the Golgi and the endosomal compartments and is important for early embryonic development (40). This CNV may therefore deregulate vesicle cycling and endocytosis in both neurons and in bone.

***CNVs of unknown significance***

In six cases, the CNV arised *de novo* in the patient; however, this does not reflect the real number of de novo CNVs but instead reflects the unavailability of parent’s samples to test inheritance. Several patients presented more than one CNV (6 patients with 2, 1 patient with 3 and 3 patients with 4). In these cases, in which the patient had CNVs inherited from both parents, there is the question of whether these alterations together could lead to the disease in the child through epistatic effects.

**Table S1** – Patients with altered aCGH results (i.e. with CNVs classified as non-polymorphic).

|  | **Clinical cohort (CC)** | **Research cohort (RC)** | **Both cohorts** |
| --- | --- | --- | --- |
| Number of patients | 137 | 188 | 325 |
| Pathogenic | 12 (8.8%) | 14 (7.4%) | 26 (8%) |
| Likely pathogenic | 8 (5.8%) | 9 (4.8%) | 17 (5.2%) |
| VOUS | 24 (17.5%) | 31 (16.5%) | 55 (16.9%) |
| Total with CNVs | **44 (32.1%)** | **54 (28.7%)** | **98 (30.1%)** |

Table S2 – List of variants of unknown clinical significance (VOUS)

| **Patients** | **Gender** | **Alteration (Hg19)** | **Type** | **Size (Kb)** | **Genes (nº)** | **Genes (name)** | **Confirmation** | **Inheritance** | **DGV controls** | **Similar case (Decipher)** | **Array platform** |
| --- | --- | --- | --- | --- | --- | --- | --- | --- | --- | --- | --- |
| R24 | Male | 1p13.2(112,243,130-112,331,235)X3 | dup | 88 | 8 | *AK023457, AK092511, BC041890, C1orf183, DDX20, DP103, KCND3, RAP1A* | qPCR | *de novo* | No | No | 1 |
| R25 | Male | 1p34.3(36,775,225-369,17,965)X3 | dup | 143 | 8 | *LSM10, OSCP1, SH3D21, STK40* | NP | ND | 1/270 (del) | No | 1 |
| C21 | Female | 1q44(245,132,097-245,259,567)x3 | dup | 127.5 | 1 | *EFCAB2* | NP | ND | 6/46505 | 265207 | 2 |
| C22,C23,C24§ | Male, Male, Male | 2p15(61,377,041-61,522,171)x3mat | dup | 14 | 3 | *C2orf74; AHSA2; USP34* | qPCR | Maternal | No | 256542; 279248 (del) | 2 |
| R26 | Male | 2q11.2(96,735,183-98,228,265)X3mat | dup | 1496 | 24 | *ARID5A, NEURL3, SEMA4C* | NP | Maternal¥ | 1/6533 | 254924,  274288 | 1 |
| C25 | Male | 2q12.3q13(109,269,051-110,504,320)x3 | dup | 1240 | 7+2 | *LIMS1 (PINCH); RANBP2; EDAR; CCDC138; SH3RF3; SEPT10; SOWAHC; MIR4265; SH3RF3-AS1* | NP | ND | No | 263424 | 3 |
| C26 | Male | 2q21.1(131,592,472-131,886,566)x1 | del | 294.1 | 3 | *ARHGEF4; FAM168B (MANI)* | qPCR | Maternal¥ | 40/52795 | 2311, 253247, 263742, 281771, 284907(all slightly bigger) | 2 |
| C27 | Female | 2q31.2q31.3(179,933,642-180,709,664)x3 | dup | 776 | 2 | *SESTD1; ZNF385B* | NP | Paternal¥ | No | No | 3 |
| R27 | Female | 2q33.1(203,338,376-203,513,494)X3 | dup | 175 | 2 | *BMPR2, FAM117B* | NP | ND | No | No | 1 |
| C28 | Male | 3q23(141,021,128-141,154,103)x3 | dup | 130 | 1 | *ZBTB38 (CIBZ)* | NP | ND | 2/18978 (smaller) | No | 3 |
| R28 | Male | 3q26.33(181,357,672-181,466,211)X1 | del | 108 | 2 | *SOX2; SOX2OT* | qPCR | *de novo* | No | 301549 (smaller dup) | 1 |
| C29 | Female | 4p16.2(5,468,286-5,812,963)x1 | del | 344.7 | 4 | *EVC2; EVC; STK32B; C4orf6* | NP | ND | 1/17421 (slightly bigger) | No | 2 |
| C30 | Male | 5q11.2(54,433,299-57,129,848)x3 | dup | 2607 | 20 | *CDC20B; GPX8; MIR449A; MIR449B; MIR449C; CCNO; DHX29; SKIV2L2; PPAP2A; RNF138P1; SLC38A9; DDX4; IL31RA; IL6ST; ANKRD55; MAP3K1; C5orf35; MIER3; GPBP1; ACTBL2* | NP | Maternal¥ | No | No | 2 |
| C31 | Male | 5q23.1q23.2(119,470,295-121,553,124)x1 | del | 2100 | 6 | *PPR16; FTMT; ZNF474; SRFBP1; LOX; LOC100505841* | NP | ND | Smaller CNVs; none comprising all the genes | No | 3 |
| C32 | Male | 5q31.1(132,540,588-132,890,257)x3 | dup | 349.7 | 2 | *FSTL4; MIR1289* | NP | ND | 1/17421 | No | 3 |
| C33 | Male | 6p22.3(18,596,430-22,732,054)x1 | del | 4136 | 7+2 | *CDKAL1 ; ID4; SOX4; E2F3; PRL; MBOAT1; HDGFL1; LOC729177; LINC00340* | NP | Maternal¥ | No | 249613 | 2 |
| R29 | Male | 7q11.23(72,741,861-73,145,916)x1mat^‡^ | del | 0.4 | 11 | *BAZ1B, STX1A, WBSCR22* | qPCR | Maternal | No | No | 1 |
| C34 | Male | 7q36.1(151,768,386-152,077,451)x3 | dup | 309.1 | 2 | *KMT2C (MLL3); GALNT11* | NP | Paternal¥ | 4/29957 | 248263 | 3 |
| C35 | Male | 8p23.1(9,687,615-10,112,430)x3 | dup | 424.8 | 3 | *LINC00599; MIR124; MSRA* | NP | Paternal¥ | No | No | 2 |
| R30 | Male | 9p24.1(6,668,082-7,112,536)X1 | del | 444 | 1 | *KDM4C* | NP | ND | 2/ 29084 | 303548  (smaller) | 1 |
| R31 | Male | 10p11.23(30,659,736-30,761,192)X3 | dup | 101 | 7 | *MAP3K8* | qPCR | Paternal¥ | 1/17369 | No | 1 |
| R32 | Male | 10q24.2(100,014,985-100,100,985)X3 | dup | 86 | 1 | *LOXL4* | NP | ND | No | No | 1 |
| R33 | Male | 11p11.2(44,601,486-44,779,120)X3 | dup | 178 | 1 | *CD82* | NP | ND | No | No | 1 |
| R34 | Male | 11q23.3(119,415,826-119,560,414)X3 | dup | 145 | 1 | *PVRL1* | NP | ND | No | No | 1 |
| R35 | Male | 12p13.33p13.32(2,248,863-3,497,525)X3pat | dup | 1248 | 9 | *CACNA1C, TULP3, FOXM1, TSPAN9* | qPCR | Paternal | 2/29084 (smaller) | No | 1 |
| R36 | Male | 13q32.3(99,451,824-99,530,240)X3 | dup | 78 | 3 | *DOCK9* | NP | ND | 1/29084  1/3017  1/10 | No | 1 |
| R37 | Male | 14q24.2(71,814,635-71,927,259)X3 | dup | 113 | 1 | *SNORD56B* | NP | ND | No | No | 1 |
| C36 | Male | 14q32.32-q32.33(103,997,076-105,608,966)x3 | dup | 1610 | 27 | *TRMT61A; BAG5; C14orf153; KLC1, XRCC3, ZFYVE21, PPP1R13B, C14orf2, TDRD9, ASPG, MIR203, KIF26A, C14orf180, TMEM179, INF2, ADSSL1, SIVA1, AKT1, ZBTB42, MGC23270, KIAA0284, PLD4, AHNAK2, C14orf79, CDCA4, GPR132, JAG2* | NP | Paternal¥ | 1/29084 (del bigger) | UOM272256 but has additional alterations | 2 |
| C2 | Male | 15q13.3(31,985,493-32,443,078)x3 | dup | 457.8 | 2 | *CHRNA7, OTUD7A* | NP | ND | 4/181  25/771  1/17421  37/29084 | 248307, 248334, 257139, 262435, 262984, 337131 | 2 |
| C37 | Male | 15q26.3(100,269,795-100,956,135)x3 | dup | 686 | 5 | *LYSMD4, DNM1P46,*  *ADAMTS17, FLJ42289, CERS3* | NP | Paternal¥ | 1/29084 (bigger; several smaller dups) | 331128 | 4 |
| C38 | Fenale | 16q11.2q12.1(46,906,585-47,199,337)x3 | dup | 292.8 | 4 | *GPT2, DNAJA2, ITFG1, NETO2* | NP | *de novo* | 1/29084 (bigger) | No | 3 |
| C39 | Male | 17p13.3(2,287,362-2,287,555)x3 | dup | 194 | 1 | *MNT* | NP | ND | 5/46874 (bigger) | No | 2 |
| R38 | Male | 17p13.1(6,955,115-7,409,331)X1 | del | 454 | 30 | *DLG4, GABARAP, DULLARD, NEURL4, NLGN2, CHRNB1* | NP | *de novo* | 3/19159 (0,000052) | 260507,  2346,  3474 all smaller del) | 1 |
| R39 | Female | 17q11.2(27,429,294-27,516,778)X3 | dup | 87 | 1 | *MYO18A* | qPCR | Paternal¥ | No | No | 1 |
| R40 | Male | 17q21.31(43,696,388-43,979,132)X3 | dup | 283 | 7 | *C17orf69, CRHR1, IMP5, LOC100128977, LOC100130148, MAPT, MGC57346* | NP | ND | 2/17421  (smaller) | No | 1 |
| R41 | Male | 19p13.2(7,077,066-7,727,437)X3 | dup | 605 | 14 | *KHSRP, PSPN, TUBB4, STXBP2* | NP | Maternal | 7/29855  (0,00023) | 253443 | 1 |
|  |  | 19p13.3-p13.2(6,332,716-6,993,284)X3 | dup | 660 | 22 | *PNPLA6, ARHGEF18, KIAA1543* | NP | Maternal | 3/181 (smaller; half the size) | No |  |
| R42 | Female | 19q13.12(37,775,477-37,942,465)X3 | dup | 167 | 3 | *HKR1, ZNF527, ZNF569* | NP | ND | 1/270 (del) | No | 1 |
| C40 | Male | 19q13.43(56,549,717-57,146,408)x3 | dup | 596.7 | 14 | *NLRP5, ZNF787,ZNF444, GALP, ZSCAN5B, ZSCAN5A, ZNF542, ZNF582, ZNF583, ZNF667, ZNF471, ZFP28, ZNF470, ZNF71* | NP | Maternal¥ | No | 259335 | 2 |
| R43 | Male | 19q13.43(58,443,388-58,669,835)X3 | dup | 226 | 8 | *C19orf18, ZNF135, ZNF256, ZNF329, ZNF418, ZNF606, ZSCAN1, ZSCAN18* | NP | *de novo* | 1/29084 | 289634 | 1 |
| R44 | Female | Xp11.21-p11.1(56,304,820-56,964,968)X3 | dup | 660 | 4 | *KLF8, UBQLN2* | NP | ND | No | 274061  (half) | 1 |
| C41 | Female | Xp21.1(32,826,352-33,936,518)x3 | dup | 1110 | 1 | *DMD* | NP | Maternal¥ | No | No | 3 |
| C42 | Female | Xp22.31(6,440,776-8,135,568)x3 | dup | 1.695 | 7 | *VCX3A, HDHD1, STS, VCX, PNPLA4* | qPCR | ND | 1/873 (smaller) | Several | 4 |
| C43 | Female | Xp22.33(480,164-785,059)x3 | dup | 304.9 | 1 | *SHOX* | NP | ND | No | 279033 (but has more variants) | 3 |
| R45 | Male | Xq28(153,230,586-153,282,378)X2 | dup | 52 | 3 | *HCFC1, IRAK1* | NP | ND | 1/265 (del) | No | 1 |
| C44 | Female | 4p16.1(8,080,960-8,416,608)x3 | dup | 335.6 | 4 | *ABLIM2; SH3TC1; HTRA3; ACOX3* | NP | ND | 1/29084 | No | 2 |
|  |  | 12p13.33(2,802,013-3,123,690)x3 | dup | 321.7 | 8 | *CACNA1C; FKBP4; ITFG2; NRIP2; FOXM1; C12orf32; TULP3;TEAD4* | NP | ND | Smaller CNVs; none comprising all the genes | UOM272277 |  |
| R46 | Male | 5q23.3(128,758,178-129,350,165)X3 | dup | 592 | 2 | *ADAMTS19, CHSY3* | NP | ND | 2/18530 (0,0001) | No | 1 |
|  |  | 9q33.3(128,474,074-128,515,941)X1 | del | 419 | 1 | *PBX3* | NP | ND | No | No | 1 |
| R47 | Male | 6q21(108,431,203-108,722,841)X3 | dup | 291 | 5 | *AF520419, LACE1, NR2E1, SNX 3A, SNX3* | NP | Maternal¥ | No | No | 1 |
|  |  | 9p24.1(6,802,781-6,943,275)X1 | del | 140 | 3 | *JMJD2C, KDM4C, KIAA0780* | NP | Paternal | 2/29084 | No | 1 |
|  |  | 16q24.3(89,867,584-89,916,614)X3 | dup | 49 | 2 | *FANCA, SPIRE2* | NP | Maternal¥ | 2/18978 (0,0001) | 248891) | 1 |
| R48 | Male | 3p21.31(48,464,967-48,574,235)X3 | dup | 109 | 7 | *ATRIP, CCDC51, CCDC72, PFKFB4, PLXNB1, SHISA5, TREX1* | NP | Maternal¥ | 1/ 17421 (del)  1/2026 (del) | No | 1 |
|  |  | 7p22.3(1,565,982-1,701,871)X3 | dup | 136 | 5 | *KIAA1908, MAFK, PSMG3, TFAMP1, TMEM184A* | NP | Paternal | 1/270 (del) | No | 1 |
|  |  | 10p12.31(20,641,191-21,122,699)X3 | dup | 481 | 2 | *NEBL, PLXDC2* | NP | Maternal¥ | No | 250836 (smaller) | 1 |
|  |  | 14q31.3(88,794,387-88,853,440)X3 | dup | 59 | 1 | *SPATA7* | NP | *de novo* | No | No | 1 |
| R49 | Female | 1p22.1(92753417-92916646)X3 | dup | 163 | 2 | *GLMN, RPAP2* | NP | Paternal ¥ | 1/ 17421 (smaller) | 284999, 276287 (partially overlap) | 1 |
|  |  | 8q21.11(76,470,859-77,036,939)X3 | dup | 566 | 1 | *HNF4G* | NP | Maternal ¥ | 1/ 17421 (smaller)  1/3017 (0,0003) | No | 1 |
|  |  | 9q21.13(78,311,144-78,695,190)X1 | del | 384 | 2 | *PCSK5* | NP | Paternal ¥ | No | No | 1 |
|  |  | 15q21.3(57,639,792-58,142,922)X3 | dup | 503 | 4 | *CGNL1, GRINL1A* | NP | Maternal ¥ | 2/29084 (0,00007) | No | 1 |
| R50 | Male | 18q12.1(29,316,291-29,569,853)X1 | del | 254 | 2 | *TRAPPC8, SLC25A52* | NP | Paternal ¥ | No | No | 1 |
|  |  | 20q11.23(36,531,120-36,618,758)X3 | dup | 88 | 2 | *VSTM2L* | NP | *de novo* | 1/29084 (del) | No | 1 |
| R51 | Female | 5p15.1(16,112,927-16,260,219)X3 | dup | 147 | 1 | *MARCH11* | NP | ND | No | 289370 | 1 |
|  |  | 13q31.1(84,644,861-84,723,563)X3 | dup | 79 | 1 | *MIR548F1* | NP | ND | 1/29084 (0,00003) | No | 1 |
| R52 | Male | 9q34.3(137,932,744-138,316,317)X3 | dup | 383 | 1 | *OLFM1* | NP | ND | 1/29084; 1/17421 | 299027 (smaller) | 1 |
|  |  | Xq28(153,130,545-153,282,378)X2 | dup | 151 | 8 | *ARHGAP4, AVPR2, HCFC1, IRAK1, L1CAM, NAA10, RENBP, TMEM187* | NP | ND | 1/265 (del) | 323738 | 1 |
| R53 | Female | 10q25.3(118,404,726-119,052,432)X3 | dup | 647 | 7 | *C10orf82, HSPA12A, KCNK18, KIAA1598, PDZD8, SLC18A2, VAX1* | NP | ND | No | No | 1 |
|  |  | 10q26.11(119,297,989-119,351,151)X3 | dup | 53 | 2 | *EMX2, EMX2OS* | NP | ND | 1/ 29084; 1/2026 (smaller) | No | 1 |
|  |  | Xp22.33(1,549,311-1,641,335)X3 | dup | 92 | 2 | *ASMTL, P2RY8* | NP | ND | 1/2 (del) | 306493, 288916, 288492, 288579 | 1 |
| R54 | Female | 11q23.3(119,415,826-119,546,072)X3 | dup | 130 | 1 | *PVRL1* | NP | ND | No | No | 1 |
|  |  | 13q31.3(92,352,559-92,668,273)X1 | del | 315 | 1 | *GPC5* | NP | ND | 1/ 17421 (smaller)  1/3017 (smaller) | 301689 | 1 |

Legend: **Patients R24 to R54: from research cohort; Patients C21 to C44: from clinical cohort; dup: duplication; del: deletion; NP: not performed; ND: not determined; .Array platform 1: Agilent 180K; 2: KaryoArray®v3.0 (Agilent 8x60k); 3: Affymetrix CytoScan HD array; 4: Affymetrix CytoScan 750K; ¥: presumably healthy; §: siblings; Cut-off for inclusion of a CNV in this table: <1/5000 in DGV.**

**PART 2 – Detailed methodology**

*aCGH*

aCGH was performed using the following platforms: Agilent 180K (AMADID:023363; 180.000 in situ synthesized 60-mer oligonucleotide probes, mean resolution of 17Kb); KaryoArray®v3.0 (Agilent 8x60k) (probes distributed throughout the genome with an average resolution of 9Kb in 357 regions associated with microdeletion/microduplication syndromes, telomeres and centromeres, and with an average resolution of 175Kb in the backbone); Agilent Whole Genome 244K (240.000 markers distributed throughout the genome, with an average resolution of 9Kb); Affymetrix CytoScan HD (probes distributed throughout the genome, with an average resolution of 20Kb) or CytoScan 750K (750.000 markers distributed throughout the genome, with a medium resolution of 8-20Kb). A diploid DNA without variations was used as a reference: for the Agilent 180K (Kreatech´s MegaPoll Reference DNA, Kreatech Diagnostics, Amsterdam); for other Agilent platforms: according to manufacturer instructions; for the Affymetrix platforms: diploid genomic DNA provided with the CytoScan® Array Kit. Genomic coordinates are according to Human Genome Build hg19; analysis was performed using the appropriate software of each platform: Agilent 180K (GEO accession number GPL15397), Nexus Copy Number 6.0 software with FASST2 Segmentation algorithm (BioDiscovery Inc, El Segundo, CA); KaryoArray®v3.0 (Agilent 8x60k) and Agilent Whole Genome 244K, Aberration Detection Method 2 (ADM-2); Affymetrix CytoScan HD and CytoScan 750k, Analysis Suite (ChAS 3.0) software (Affymetrix).

*CNV classification criteria*

The genomic variants detected were classified using adapted criteria, previously described elsewhere (41–44).

A CNV was classified as pathogenic when a high degree of certainty of their clinically significance is present in the literature. This group includes patients carrying large CNVs that overlap significantly with a region with an established pathogenic effect. This classification was also applied to susceptibility *loci* of variable expressivity and incomplete penetrance (reviewed in (45)).

Likely pathogenic variants include newly described gene rich large CNVs that comprise good candidates for disease association. In this category were included only CNVs whose speculation of pathogenicity is supported in the literature (for example, there is another similar patient described or it includes gene(s) with a compelling function). However, the uncertainty of this claim stills remains. It is possible that variants in this category will be latter classified as pathogenic (with the report of other similar CNVs in patients with overlapping phenotype) or as benign (for example, if the variant is described later on in several unaffected cases).

Variants of unknown clinical significance (VOUS) include variants whose clinical significance is not yet possible to speculate, due to the fact that: 1) there is a lack of overlapping CNVs reported in the literature and/or databases; 2) the CNV contain genes but it is not yet known whether they are dosage sensitive; 3) the CNV is described in multiple contradictory publications and/or databases, and firm conclusions regarding clinical significance are not yet established (44).

*Quantitative PCR confirmations*

Quantitative PCR reactions were carried out in a 7500-FAST Real Time PCR machine

(Thermo Fisher Scientific, Waltham, MA, USA) using Power SYBR Green® (Thermo Fisher Scientific, Waltham, MA, USA), as described elsewhere (46) and following the general recommendations for qPCR (47,48). The specificity of each reaction was verified by the generation of a melting curve for each of the amplified fragments. The primer efficiency was calculated by generation of a standard curve fitting the accepted normal efficiency percentage (primers used for all genes are listed in table S3). Ct values ​​obtained for each test were analyzed in DataAssist™ software (Thermo Fisher Scientific, Waltham, MA, USA).

*mRNA expression analysis*

Total RNA was isolated from leucocytes of patients and controls (ten healthy controls, five females and five males) using QIAsymphony RNA Kit (QIAGEN GmbH, Germany), according to the manufacturers’ protocol. First-strand cDNA, synthesized using SuperScript® III Reverse Transcriptase (RT) (Thermo Fisher Scientific, Waltham, MA USA). The genes selected to study within each CNV were selected based on their localization in the alteration (either in breakpoint or close by), their functional relevance and their predicted expression values in the periphery – genes without or with very low peripheral expression were not analyzed [data retrieved from GeneCards database ([www.genecards.org](http://www.genecards.org/)) and GTExportal ([http://www.gtexportal.org](http://www.gtexportal.org/) )]. Primers used for all genes are listed on table S4. Quantitative PCR reactions were carried out in a 7500-FAST Real Time PCR machine (Thermo Fisher Scientific, Waltham, MA, USA) using Power SYBR Green® (Thermo Fisher Scientific, Waltham, MA, USA). The expression levels of the genes were normalized to the *B2M*, B-ACTIN, *TRAP1* or *PPIB* genes and relative quantification was used to determine the fold change difference between each gene and each reference gene, using the DDCT method, as described elsewhere and following the general recommendations (49,50).

*Methylation status*

Methylation status for *SNRPN* gene (*locus* 15q11-13) was studied by MLPA for patient C10 using the MLPA kit ME028-C1 (MRC Holland) in accordance with manufacturer instructions.

**Table S3** – Primers used for quantitative PCR confirmation.

| **Chromosome** | **Gene** | **Reference sequence** | **Primer location** | **Primer Forward 5'🡪3'** | **Primer Reverse 5'🡪3'** | **Amplicon size (bp)** |
| --- | --- | --- | --- | --- | --- | --- |
| Chr 1 | *AKT3* | NM_005465.4 | Exon7 | TCTGGGCTTAACCTCTTCCA | TGTTAAAAAGGGATGTCTAGTGTTC | 162bp |
| Chr 1 | *AKT3* | NM_005465.4 | Exon8 | CCTTGAAATATTCCTTCCAGACA | CCATGCAAATACTGGATTTACTTCT | 101bp |
| Chr 1 | *AKT3* | NM_005465.4 | Exon9 | AGAGAGCGGGTGTTCTCTGA | CCTTGAGATCACGGTACACAA | 106bp |
| Chr 1 | *AKT3* | NM_005465.4 | Exon10 | CAGTTGGAGAATCTAATGCTGGA | AATGGAACCGAAGCCTACCT | 150bp |
| Chr 1 | *MAST2* | NM_015112.2 | Exon3 | AGCTGCTCCCTTTGTCCAG | GCCACCTTTATGAACACTTACCAG | 158bp |
| Chr 1 | *PRKAB2* | NM_005399.4 | Exon4 | AGCCATAATGACTTTGTTGCCA | GCCCATCAGTCTTGACAGAAA | 174bp |
| Chr 1 | *FAM69A* | NM_001006605.4 | Exon3 | AGACTGGAGTTATTGATGGGC | CTGGAATGTTTATTCATAATGGC | 130bp |
| Chr 2 | *GPR45* | NM_007227.3 | Exon1 | ACGTCCCTTGAGGCTTACAC | ACGATGATGCAGACCACAGT | 161bp |
| Chr 2 | *ARHGEF4* | NM_015320.3 | Exon14 | TTCTGGCACAGCATCAGC | CACTTGCAGGCAGAGGAAG | 144bp |
| Chr 2 | *USP34* | NM_014709.3 | Exon80 | ATGAAGGAGCAACTCCCATT | GCTCAGTTCCTGGATCAATAAT | 168bp |
| Chr 3 | *SOX2* | NM_003106.3 | Exon1 | CCCACCTACAGCATGTCCTA | CTGATCATGTCCCGGAGGT | 164bp |
| Chr 3 | *ZNF80* | ENSG00000174255 | Exon1 | GCTACCGCCAGATTCACACT | AATCTTCATGTGCCGGGTTA | 182bp |
| Chr 7 | *CNOT4* | NM_001190850.1 | Exon10 | CACCGAGCGGTTTATAATTCA | AGACCTGTGTTGTGCTGTGG | 164bp |
| Chr 7 | *OCM* | NM_001097622.1 | Intron1/exon2 | ctctgttcttcagACCCAGACA | gcttacTTAAGCTCTTCTTCATCCA | 152bp |
| Chr 7 | *CALD1* | NM_033139.3 | Exon4 | GAATGACGATGATGAAGAGGAG | ACAGTACCTGTTCTGGGCATTC | 139bp |
| Chr 7 | *BAZ1B* | NM_032408.3 | Exon3 | TCCTGCCTGGTATGAGAAGC | TCCCACAGCATATTTGGTCA | 112bp |
| Chr 9 | *LHX2* | NM_004789.3 | Exon3 | GCTCGGGACTTGGTTTATCA | GTTGAAGTGTGCGGGGTACT | 156bp |
| Chr 9 | *ZNF658* | NM_001317916.1 | 5’UTR | ACCTCTTTGGTATAAACGTTCCAT | AGGACAGGGAGTCACATCTCTC | 119bp |
| Chr 10 | *MAP3K8* | NM_005204.3 | Exon3 | TGGAGTACATGAGCACTGGAA | TTGACACATGGTCATTAGACTGG | 152bp |
| Chr 10 | *EBF3* | NM_001005463.2 | Intron15 | CTCTCTGCTGGGTGCTGAG | GCGTCCCTTCATACGCTAAC | 169bp |
| Chr 11 | *KIRREL3* | NM_032531.3 | Exon17/3’UTR | GATGCAGACTCACGTCTAAGGA | CTTGATCAGAGCTTCGAAGGAA | 179bp |
| Chr 12 | *TULP3* | NM_003324.4 | Exon3 | GGCTACTACTTGAGAAGAGGCAAA | TGACATTGCTGTGGGGAGTA | 150bp |
| Chr 12 | *MED13L* | NM_015335.4 | Exon3/Intron3 | GGAAGAAGGACTCTGGGAAAA | CAGGAAACTCTCGGTATCTAGCA | 151bp |
| Chr 15 | *SNRPN* | NM_003097.3 | 5’UTR | CTTTCCTGTCTGTCATTTTGC | GTCCCTTCTCTGTGCAGC | 160bp |
| Chr 17 | *MYO18A* | NM_00134765.1 | Exon2 | TGTCAAGCGCTTTTCCTTCT | AGAGTCCTCACCTCCACCTG | 111bp |
| Chr 20 | *SDC4* | ENSG00000124145 | Exon4 | ACCGAACCCAAGAAACTAGA | GTGCTGGACATTGACACCT | 101bp |
| Chr 20 | *EBF4* | NM_001110514.1 | Exon16 | GCTGCCTCCTCCATGTCC | AAGGCGCTCCTCTGTTTGAC | 101bp |
| Chr X | *ARHGEF6* | NM_004840.2 | 3’UTR | CTTGAAATGTCCCGCTGAAT | AACAACAGCAAATGCCCAAG | 162bp |
| Chr X | *PNPLA4* | NM_001142389.1 | Exon6 | CACCAACGCTCTTCCCAT | CACCATGATATCCTGCTTGG | 136bp |
| Chr X | *CUL4B* | NM_003588.3 | Exon3 | CTTCAACCTCGTCCTTCTGC | GTTGCAGCAGTTGGTGAAGA | 166bp |
| Chr X | *CUL4B* | NM_003588.3 | Exon21 | ATTGATGCTGCAATTGTTCG | TGTTTGCAAGATTTGTGTCTGA | 182bp |
| Chr X | *HUWE1* | NM_031407.5 | Exon69 | TGTTGACATCCCACTCTTGTTC | TTGTTTACAAAGGGTATAACCCAGA | 152bp |
| Chr X | *HUWE1* | NM_031407.5 | Exon75 | GGCACACATCAAGGACGAG | GCAAAGCGAAGGAACTTCTG | 153bp |

**Table S4 – Primers used for expression studies.**

| **Chromosome** | **Gene** | **Reference sequence** | **Primer Forward 5'🡪3'** | **Primer location** | **Primer Reverse 5'🡪3'** | **Primer location** | **Amplicon size (bp)** |
| --- | --- | --- | --- | --- | --- | --- | --- |
| Chr 1 | *FAM69A* | NM_001006605.4 | AGACTGGAGTTATTGATGGGC | Exon3 | CACAACACCTGGTAGATTATCCC | Exon4 | 134bp |
| Chr 1 | *DPYD* | [NM_000110.3](https://www.ncbi.nlm.nih.gov/nuccore/NM_000110.3) | GCAGCAATTTGCTACTGAGG | Exon5 | CCCAGCACCAAAAAGAGC | Exon6 | 123bp |
| Chr 1 | *TGFBR3* | [NM_003243.4](https://www.ncbi.nlm.nih.gov/nuccore/NM_003243.4) | GCCTTGATGGAGAGCTTCAC | Exon3 | GGGATTCAGGTGAAGTGTGAC | Exon4 | 146bp |
| Chr 5 | *PPIB* | NM_000942.4 | TGACCTACGAATTGGAGATGAAG | Exon2 | TGCTGTTTTTGTAGCCAAATCCT | Exon3 | 130bp |
| Chr 7 | *CALD1* | NM_033139.3 | GCAGAAAAGCAGTGGTGTCA | Exons8/9 | CCTTCAGCAGGAACAGGAAG | Exon10 | 152bp |
| Chr 7 | *AGBL3* | NM_178563.3 | TCCATTGACTCTCTGACTTACCTTC | Exon12 | ATCTGGTTCATTTGGCCTTG | Exon15 | 194bp |
| Chr 7 | *CNOT4* | NM_001190850.1 | CCTGCATGTAGAAAGCCATATCC | Exons2/3 | GTACACTAGCCAAATGTTTGCG | Exon3 | 150bp |
| Chr 7 | *EXOC4* | NM_021807.3 | CACTACACAGAATTGACGACAGC | Exon2 | TTTCCGAAGCTCATCCCGTTT | Exon3 | 146bp |
| Chr 9 | *EHMT1* | NM_024757.4 | CTGCATGCAGCCAGTAAAGATC | Exons3/4 | CTGCTGTCGTCCAAAGTCAG | Exon4 | 104bp |
| Chr 9 | *CACNA1B* | NM_000718.3 | TGGTGTCTGGGATTCCAAG | Exon4 | CCATGTAGAACTCCAGGCCA | Exon5 | 134bp |
| Chr 9 | *TSC1* | NM_000368.4 | GATAGAACTGAAGAAGGCCAAC | Exon19 | GTGCTTGTTCTGCAGTTGTTCC | Exon20 | 177bp |
| Chr 9 | *FBXW2* | NM_012164.3 | CTTGTGACAGGCTCCTTTGAC | Exon4 | ATTGTAGTCCACGCTAAATACCG | Exons4/5 | 111bp |
| Chr 9 | *NEK6* | NM_001145001.2 | AAGATAGGCCGAGGACAGTTC | Exon4 | CCATCATCTCAAAGATCTG | Exons4/5 | 99bp |
| Chr 9 | *PSMB7* | NM_002799.2 | TTTCTCCGCCCATACACAGTG | Exon7 | AGCACCTCAATCTCCAGAGGA | Exon8 | 119bp |
| Chr 12 | *TSPAN9* | NM_006675.4 | AACATCATCCAGGCTGAG | Exon6 | GAGTTCTCCATGCAGCAG | Exon7 | 106bp |
| Chr 14 | *TECPR2* | NM_014844.3 | GGGGAAGACGGAATCTATCA | Exons2/3 | GTGACATCAAATCTCCGAAGCT | Exons3/4 | 149bp |
| Chr 14 | *INF2* | NM_022489.3 | GACCACTTCTACCTCCTCCTG | Exon12 | TGAGGAAGTTCCCAATTCTC | Exon14 | 201bp |
| Chr 15 | *B2M* | NM_004048 | GATAGTTAAGTGGGATCGAG | Exon 2/3 | GCAAGCAAGCAGAATTTGGA | Exon4 | 93bp |
| Chr X | *LAMP2* | NM_002294.2 | ACCACTGTGCCATCTCCTAC | Exon5 | GAGTCTAAGTAGAGCAGTGTGAG | Exon6 | 215bp |
| Chr X | *CUL4B* | NM_003588.3 | GCATTCTTCTCTTGATTGAGAGG | Exon8 | GAGCCGGTTAGTTTCTTCC | Exon9 | 142bp |
| Chr X | *FHL1* | NM_001159702.2 | AAGAACCGCTTCTGGCATGAC | Exon4 | CCCCTTGTACTCCACGTTTTG | Exon5 | 188bp |
| Chr X | *ARHGEF6* | NM_004840.2 | TCCTCGCTGAAAAATGGGGTA | Exon1 | CTTGGAGGGTTGCACATCCT | Exon2 | 147bp |
| Chr X | *MAP7D3* | NM_024597.3 | TTGTCATCTGCAGGCCTTC | Exon6 | GCATTACATAATTGGTGACGC | Exon8 | 159bp |

### PART 3 – Genes within likely pathogenic CNVs: data retrieved from OMIM, Decipher, ClinVar and ExAC databases (6,51)

**Table S5 - OMIM entrance, haploinsufficiency score and constrain metrics for the selected genes in patient R16.**

| **2q11.2-q12.2 deletion** | **List of all the**  **genes affected** | ***C2orf29, C2orf49, CREG2, FHL2, GPR45, IL18R1, IL18RAP, IL1R1, IL1R2, IL1RL1, IL1RL2, LOC150568, MAP4K4, MFSD9, MRPS9, POU3F3, RFX8, RNF149, SLC9A2, SLC9A4,***  ***SNORD89, TBC1D8, TGFBRAP1, TMEM182*** | | | | | | | |
| --- | --- | --- | --- | --- | --- | --- | --- | --- | --- |
| **Gene** | **Morbid gene** | **OMIM** | **% HI score** | **DDG2P** | **ClinVar** | **Constraint Metrics** | | | |
|  |  |  |  |  |  | **Synonymous (z)** | **Missense (z)** | **LoF (pLI)** | **CNV (z)** |
| *MAP4K4* | No | - | 20-30% | - | 1del/7dups | -0.83 | 4.01 | 1 | 0.19 |
| *FHL2* | No | - | 20-30% | - | 4dels/7dups/32SNVs | -0.15 | 0.35 | 0 | 0.53 |
| *POU3F3* | No | - | 20-30% | - | 4dels/6dups | ND | ND | ND | ND |
| *CNOT11* | No | - | 20-30% | - | 2dels9dups | 1.66 | 3.76 | 0.99 | 0.18 |
| OMIM: Online Mendelian Inheritance in Man; HI score: Haploinsufficiency Score index - high ranks (e.g. 0-10%) indicate a gene is more likely to exhibit haploinsufficiency, low ranks (e.g. 90-100%) indicate a gene is more likely to NOT exhibit haploinsufficiency (retrieved from Decipher); DDG2P: **Developmental Disorders Genotype-Phenotype Database;** LoF: Loss of function; CNVs: copy number variations; z: Z score is the deviation of observed counts from the expected number for one gene (positive Z scores = gene intolerance to variation, negative Z scores = gene tolerant to variation) (retrieved from ExAC); pLI: probability that a given gene is intolerant of loss-of-function variation (pLI closer to one = more intolerant the gene is to LoF variants, pLI >= 0.9 is extremely LoF intolerant) (retrieved from ExAC); del – deletion; dup – duplication; SNV – single nucleotide variant; ins – insertion; indel – insertion/deletion. | | | | | | | | | |

**Table S6 -OMIM entrance, haploinsufficiency score and constrain metrics for the selected genes in patient C15.**

| **17p11.2 deletions (both)** | **List of all the**  **genes affected** | ***TNFRSF13B, MPRIP, PLD6, FLCN, COPS3 + TRIM16L, ZNF286B, TBC1D28, FBXW10, TVP23B, PRPSAP2, SLC5A10, GRPA, FAM83G, GRAPL, EPN2, B9D1, MAPK7, MFAP4, RNF112,***  ***SLC47A1, ALDH3A2, SLC47A2, ALDH3A1, ULK2, AKAP10, SPECC1, LGALS9B, CDRT15L2, CCDC144NL, USP22, DHRS7B, TMEM11, C17orf103, MAP2K3*** | | | | | | | |
| --- | --- | --- | --- | --- | --- | --- | --- | --- | --- |
| **Gene** | **Morbid gene** | **OMIM** | **% HI score** | **DDG2P** | **ClinVar** | **Constraint Metrics** | | | |
|  |  |  |  |  |  | **Synonymous (z)** | **Missense (z)** | **LoF (pLI)** | **CNV (z)** |
| *COPS3* | No | - | 0-10% | - | 33dels/29dups | 0.06 | 1.85 | 0.99 | 0.91 |
| *EPN2* | No |  | 40-50% | - | 27dels/28dups | 0.16 | 1.09 | 0.18 | 0.58 |
| *B9D1* | Yes | -614209, ?Meckel syndrome 9; 617120, Joubert syndrome 27 | 50-60% | Probable | 29dels/28dups/23SNVs | -0.27 | 0.20 | 0.12 | 0.42 |
| *RNF112* | No | - | 70-80% | - | 27dels/28dups | -1.07 | 1.15 | 0 | 0.46 |
| *ULK2* | No | - | 30-40% | - | 27dels/30dups/1SNV | 0.03 | 0.61 | 0 | -0.3 |
| *ALDH3A2* | Yes | 270200, Sjogren-Larsson syndrome (AR) | 50-60% | Yes | 47dels/34dups/62SNVs/2indel/7ins | 1.06 | 0.47 | 0.01 | -0.76 |
| *AKAP10* | Yes | 115080, Cardiac conduction defect, susceptibility to | 10-20% | - | 27dels/30dups/2SNVs | -0.75 | 0.38 | 0.93 | 0.41 |
| *MAP2K3* | No | - | 10-20% | - | 3dels/4dups/1SNV | -0.13 | -0.23 | 0 | -4.29 |
| *TMEM11* | No | - | 10-20% | - | 2dels/4dups | 0.22 | 2.06 | 0.78 | 0.24 |
| OMIM: Online Mendelian Inheritance in Man; HI score: Haploinsufficiency Score index - high ranks (e.g. 0-10%) indicate a gene is more likely to exhibit haploinsufficiency, low ranks (e.g. 90-100%) indicate a gene is more likely to NOT exhibit haploinsufficiency (retrieved from Decipher); DDG2P: **Developmental Disorders Genotype-Phenotype Database;** LoF: Loss of function; CNVs: copy number variations; z: Z score is the deviation of observed counts from the expected number for one gene (positive Z scores = gene intolerance to variation, negative Z scores = gene tolerant to variation) (retrieved from ExAC); pLI: probability that a given gene is intolerant of loss-of-function variation (pLI closer to one = more intolerant the gene is to LoF variants, pLI >= 0.9 is extremely LoF intolerant) (retrieved from ExAC); del – deletion; dup – duplication; SNV – single nucleotide variant; ins – insertion; indel – insertion/deletion. | | | | | | | | | |

**Table S7- OMIM entrance, haploinsufficiency score and constrain metrics for the selected genes in patient R20.**

| **20q13.12-q13.13 deletion** | **List of all the**  **genes affected** | ***ACOT8, ARFGEF2, B4GALT5, C20orf123, C20orf165, C20orf199, CD40, CDH22, CEBPB, CSE1L, CTSA, DBNDD2, DDX27, DNTTIP1, ELMO2, EYA2, KCNB1, KCNK15, KCNS1, LOC100131496, LOC100240726, LOC284749, MATN4, MIR1259, MMP9, NCOA3, NCOA5, NEURL2, PABPC1L, PCIF1, PI3, PIGT, PLTP, PREX1, PTGIS, RBPJL, RIMS4, RNF114, SDC4, SEMG1, SEMG2, SLC12A5, SLC13A3, SLC2A10, SLC35C2, SLC9A8, SLPI, SNAI1, SNORD12, SNORD12B, SNORD12C, SNX21, SPATA2, SPINLW1, SPINT3, SPINT4, STAU1, STK4, SULF2, SYS1, SYS1-DBNDD2, TMEM189, TMEM189-UBE2V1, TNNC2, TOMM34, TP53RK, TP53TG5, UBE2C, UBE2V1, WFDC10A, WFDC10B, WFDC11, WFDC12, WFDC13, WFDC2, WFDC3, WFDC5, WFDC6, WFDC8, WFDC9, WISP2, YWHAB, ZMYND8, ZNF334, ZNF335, ZNFX1, ZSWIM1, ZSWIM3*** | | | | | | | |
| --- | --- | --- | --- | --- | --- | --- | --- | --- | --- |
| **Gene** | **Morbid gene** | **OMIM** | **% HI score** | **DDG2P** | **ClinVar** | **Constraint Metrics** | | | |
|  |  |  |  |  |  | **Synonymous (z)** | **Missense (z)** | **LoF (pLI)** | **CNV (z)** |
| *KCNB1* | Yes | 616056, Epileptic encephalopathy, early infantile, 26 | 20-30% | Yes | 1del/5dups/53SNVs/1ins | 1.48 | 5.15 | 0.98 | -0.56 |
| *PIGT* | Yes | 615398, Multiple congenital anomalies-hypotonia-seizures syndrome 3; 615399, ?Paroxysmal nocturnal hemoglobinuria 2 | 30-40% | Yes | 1del/3dups/9SNV/1ins | -0.66 | -0.37 | 0 | 0.26 |
| *CTSA* | Yes | 256540, Galactosialidosis | 40-50% | Yes | 10dels/3dups/41SNVs/2indel/3ins | 1.13 | 1.45 | 0 | -0.98 |
| *SLC2A10* | Yes | 208050, Arterial tortuosity syndrome | 70-80% | Yes | 9dels/3dups/155SNVs/1ins | -0.51 | -1.01 | 0.01 | -0.48 |
| *ARFGEF2* | Yes | 608097, Periventricular heterotopia with microcephaly | 20-30% | Probable | 6dels/6dups/138SNVs/3ins | 1.18 | 2.9 | 1 | -0.04 |
| OMIM: Online Mendelian Inheritance in Man; HI score: Haploinsufficiency Score index - high ranks (e.g. 0-10%) indicate a gene is more likely to exhibit haploinsufficiency, low ranks (e.g. 90-100%) indicate a gene is more likely to NOT exhibit haploinsufficiency (retrieved from Decipher); DDG2P: **Developmental Disorders Genotype-Phenotype Database;** LoF: Loss of function; CNVs: copy number variations; z: Z score is the deviation of observed counts from the expected number for one gene (positive Z scores = gene intolerance to variation, negative Z scores = gene tolerant to variation) (retrieved from ExAC); pLI: probability that a given gene is intolerant of loss-of-function variation (pLI closer to one = more intolerant the gene is to LoF variants, pLI >= 0.9 is extremely LoF intolerant) (retrieved from ExAC); del – deletion; dup – duplication; SNV – single nucleotide variant; ins – insertion; indel – insertion/deletion. | | | | | | | | | |

**Table S8 - OMIM entrance, haploinsufficiency score and constrain metrics for the selected genes in patient C16.**

| **1p22.1-p21.3 duplication** | **List of all the**  **genes affected** | ***TGFBR3, BRDT, EPHX4, BTBD8, KIAA1107, C1orf146, GLMN, RPAP2, GFI1, EVI5, RPL5, FAM69A, SNORD21, SNORA66, MTF2, TMED5, CCDC18, LOC100131564, DR1, FNBP1L, BCAR3, LOC100129046, MIR760, DNTTIP2, GCLM, ABCA4, ARHGAP29, ABCD3, F3, SLC44A3, CNN3, LOC729970, ALG14, TMEM56, TMEM56-RWDD3, RWDD3, FLJ31662, PTBP2,***  ***DPYD, DPYD-AS1, MIR137HG, MIR2682, MIR137, LOC729987*** | | | | | | | |
| --- | --- | --- | --- | --- | --- | --- | --- | --- | --- |
| **Gene** | **Morbid gene** | **OMIM** | **% HI score** | **DDG2P** | **ClinVar** | **Constraint Metrics** | | | |
|  |  |  |  |  |  | **Synonymous (z)** | **Missense (z)** | **LoF (pLI)** | **CNV (z)** |
| *FAM69A* | No | - | 10-20% | - | 1del/3dups/16SNVs | 0.38 | 0.55 | 0.09 | nan |
| *TGFBR3* | No | - | 10-20% | - | 4dups/1SNV | -0.44 | 0.55 | 0.01 | -1.04 |
| *GLMN* | Yes | 138000, Glomuvenous malformations | 10-20% | Yes | 6dels/5dups/29SNVs/1indel/ 2ins | -1.61 | -0.18 | 0 | 0.58 |
| *EVI5* | No | - | 10-20% | - | 1del/2dups | 0.16 | -0.09 | 0 | 0.73 |
| *RPL5* | Yes | 612561, Diamond-Blackfan anemia 6 | 0-10% | - | 3dels/3dups/31SNVs/1indel/ 2ins | 0.37 | 1.52 | 0.99 | 0.61 |
| *MTF2* | No | - | 0-10% | - | 1del/2dups | -0.29 | 2.29 | 0.99 | 1.18 |
| *DR1* | No | - | 0-10% | - | 1del/2dups | -0.63 | 2.04 | 0.84 | 0.69 |
| *ABCA4* | Yes | 604116, Cone-rod dystrophy 3; 248200, Retinal dystrophy, Stargardt disease 1, Fundus flavimaculatus; 601718, Retinitis pigmentosa 19; 153800, Macular degeneration, age-related, 2 | 10-20% | - | 82dels/15dups/688SNVs/3indels/27ins | -0.74 | -1.50 | 0 | 0.12 |
| *ABCD3* | Yes | -616278, ?Bile acid synthesis defect, congenital, 5 | 10-20% | - | 2dels/3dups/4SNVs/1ins | -0.22 | 2.42 | 1 | 0.17 |
| *CNN3* | No | - | 10-20% | - | 2dups | 1.92 | 2.38 | 0.86 | 0.69 |
| *PTBP2* | No | - | 0-10% | - | 3dels/2dups | -0.26 | 3.27 | 0.99 | 0.59 |
| *DPYD* | Yes | 274270, Dihydropyrimidine dehydrogenase deficiency, 5-fluorouracil toxicity | 0-10% | - | 23dels/7dups/131SNVs/1indel/5ins | -0.68 | -1.51 | 0 | nan |
| OMIM: Online Mendelian Inheritance in Man; HI score: Haploinsufficiency Score index - high ranks (e.g. 0-10%) indicate a gene is more likely to exhibit haploinsufficiency, low ranks (e.g. 90-100%) indicate a gene is more likely to NOT exhibit haploinsufficiency (retrieved from Decipher); DDG2P: **Developmental Disorders Genotype-Phenotype Database;** LoF: Loss of function; CNVs: copy number variations; z: Z score is the deviation of observed counts from the expected number for one gene (positive Z scores = gene intolerance to variation, negative Z scores = gene tolerant to variation) (retrieved from ExAC); pLI: probability that a given gene is intolerant of loss-of-function variation (pLI closer to one = more intolerant the gene is to LoF variants, pLI >= 0.9 is extremely LoF intolerant) (retrieved from ExAC); del – deletion; dup – duplication; SNV – single nucleotide variant; ins – insertion; indel – insertion/deletion. | | | | | | | | | |

**Table S9 - OMIM entrance, haploinsufficiency score and constrain metrics for the selected genes in patient R21.**

| **9q33.2-q33.3 triplication** | **List of all the**  **genes affected** | ***C5, FBXW2, LOC253039, LOC402377, PHF19, PSMD5, TRAF1, C9orf45, CEP110, CRB2, DAB2IP, DENND1A, GGTA1, GPR21, GSN, LHX2, LHX6, LOC100129034, MIR548D1, MIR600, MIR601, MORN5, MRRF, NDUFA8, NEK6, OR1B1, OR1J1, OR1J2, OR1J4, OR1K1, OR1L1, OR1L3, OR1L4, OR1L6, OR1L8, OR1N1, OR1N2, OR1Q1, OR5C1, PDCL, PSMB7, PTGS1, RAB14, RABGAP1, RBM18, RC3H2, SNORD90, STOM, STRBP, TTLL11, ZBTB26, ZBTB6*** | | | | | | | |
| --- | --- | --- | --- | --- | --- | --- | --- | --- | --- |
| **Gene** | **Morbid gene** | **OMIM** | **% HI score** | **DDG2P** | **ClinVar** | **Constraint Metrics** | | | |
|  |  |  |  |  |  | **Synonymous (z)** | **Missense (z)** | **LoF (pLI)** | **CNV (z)** |
| *CRB2* | Yes | 616220, Focal segmental glomerulosclerosis 9; 219730, Ventriculomegaly with cystic kidney disease | 60-70% | Yes | 2dels/17dups/7SNVs/1ins | 1.14 | 1.64 | 0 | 0.84 |
| *LHX2* | No | - | 0-10% | - | 1del/16dups | 2.63 | 4.59 | 0.95 | 0.5 |
| *DENND1A* | No | - | 0-10% | - | 2dels/16dups | -0.25 | 1.31 | 0.90 | 0.80 |
| *STRBP* | No | - | 0-10% | - | 2dels/16dups | 0.39 | 3.19 | 1 | 1.02 |
| *RAB14* | No | - | 0-10% | - | 2dels/15dups | 1.01 | 2.90 | 0.97 | 1.02 |
| *GSN* | Yes | 105120, Amyloidosis, Finnish type | 0-10% | - | 1del/18dups/43SNVs/9ins | 0.78 | 1.75 | 0 | 0.51 |
| *PSMB7* | No | - | 0-10% | - | 1del/16dups | 0.05 | 0.89 | 0.94 | 0.33 |
| *LHX6* | No | - | 0-10% | - | 1del/15dups | 2.69 | 4.43 | 0.95 | 0.46 |
| *ZBTB26* | No | - | 0-10% | - | 1del/16dups | -0.93 | 1.77 | 0.02 | 0.66 |
| OMIM: Online Mendelian Inheritance in Man; HI score: Haploinsufficiency Score index - high ranks (e.g. 0-10%) indicate a gene is more likely to exhibit haploinsufficiency, low ranks (e.g. 90-100%) indicate a gene is more likely to NOT exhibit haploinsufficiency (retrieved from Decipher); DDG2P: **Developmental Disorders Genotype-Phenotype Database;** LoF: Loss of function; CNVs: copy number variations; z: Z score is the deviation of observed counts from the expected number for one gene (positive Z scores = gene intolerance to variation, negative Z scores = gene tolerant to variation) (retrieved from ExAC); pLI: probability that a given gene is intolerant of loss-of-function variation (pLI closer to one = more intolerant the gene is to LoF variants, pLI >= 0.9 is extremely LoF intolerant) (retrieved from ExAC); del – deletion; dup – duplication; SNV – single nucleotide variant; ins – insertion; indel – insertion/deletion. | | | | | | | | | |

**Table S10 - OMIM entrance, haploinsufficiency score and constrain metrics for the selected genes in patient C19**

| **9q34.3** **duplication** | **List of all the**  **genes affected** | ***EHMT1*** | | | | | | | |
| --- | --- | --- | --- | --- | --- | --- | --- | --- | --- |
| **Gene** | **Morbid gene** | **OMIM** | **% HI score** | **DDG2P** | **ClinVar** | **Constraint Metrics** | | | |
|  |  |  |  |  |  | **Synonymous (z)** | **Missense (z)** | **LoF (pLI)** | **CNV (z)** |
| *EHMT1* | Yes | 610253, Kleefstra syndrome | 60-70% | Yes | 48dels/49dups/3indels/181SNVs/9ins | 0.20 | 2.36 | 1 | -0.38 |
| OMIM: Online Mendelian Inheritance in Man; HI score: Haploinsufficiency Score index - high ranks (e.g. 0-10%) indicate a gene is more likely to exhibit haploinsufficiency, low ranks (e.g. 90-100%) indicate a gene is more likely to NOT exhibit haploinsufficiency (retrieved from Decipher); DDG2P: **Developmental Disorders Genotype-Phenotype Database;** LoF: Loss of function; CNVs: copy number variations; z: Z score is the deviation of observed counts from the expected number for one gene (positive Z scores = gene intolerance to variation, negative Z scores = gene tolerant to variation) (retrieved from ExAC); pLI: probability that a given gene is intolerant of loss-of-function variation (pLI closer to one = more intolerant the gene is to LoF variants, pLI >= 0.9 is extremely LoF intolerant) (retrieved from ExAC); del – deletion; dup – duplication; SNV – single nucleotide variant; ins – insertion; indel – insertion/deletion. | | | | | | | | | |

**Table S11 - OMIM entrance, haploinsufficiency score and constrain metrics for the selected genes in patients R22 and R23.**

| **Xq24 duplication** | **List of all the**  **genes affected** | ***C1GALT1C1, CUL4B, LAMP2, MCTS1*** | | | | | | | |
| --- | --- | --- | --- | --- | --- | --- | --- | --- | --- |
| **Gene** | **Morbid gene** | **OMIM** | **% HI score** | **DDG2P** | **ClinVar** | **Constraint Metrics** | | | |
|  |  |  |  |  |  | **Synonymous (z)** | **Missense (z)** | **LoF (pLI)** | **CNV (z)** |
| *CUL4B* | Yes | 300354, Mental retardation, X-linked, syndromic 15 (Cabezas type) | 0-10% | Yes | 56dels/48dups/20SNVs/1ins | 0.65 | 3.88 | 1 | **nan** |
| *LAMP2* | Yes | 300257, Danon disease | 50-60% | Yes | 73dels/75dups/158SNVs/13ins | 0.15 | 0.41 | 0.95 | **nan** |
| *C1GALT1C1* | Yes | 300622, Tn polyagglutination syndrome, somatic | 20-30% | - | 51dels/46dups/5SNVs | -0.78 | 0.46 | 0.69 | **nan** |
| *MCTS1* | No | - | 10-20% | - | 51dels/46dups | 0.74 | 1.86 | 0.83 | **nan** |
| OMIM: Online Mendelian Inheritance in Man; HI score: Haploinsufficiency Score index - high ranks (e.g. 0-10%) indicate a gene is more likely to exhibit haploinsufficiency, low ranks (e.g. 90-100%) indicate a gene is more likely to NOT exhibit haploinsufficiency (retrieved from Decipher); DDG2P: **Developmental Disorders Genotype-Phenotype Database;** LoF: Loss of function; CNVs: copy number variations; z: Z score is the deviation of observed counts from the expected number for one gene (positive Z scores = gene intolerance to variation, negative Z scores = gene tolerant to variation) (retrieved from ExAC); pLI: probability that a given gene is intolerant of loss-of-function variation (pLI closer to one = more intolerant the gene is to LoF variants, pLI >= 0.9 is extremely LoF intolerant) (retrieved from ExAC); del – deletion; dup – duplication; SNV – single nucleotide variant; ins – insertion; indel – insertion/deletion. | | | | | | | | | |

**Table S12 - OMIM entrance, haploinsufficiency score and constrain metrics for the selected genes in patient C20.**

| **Xq26.3 duplication** | **List of all the**  **genes affected** | ***FHL1, MAP7D3, GPR112, BRS3, HTATSF1, VGLL1, MIR934, CD40LG, ARHGEF6*** | | | | | | | |
| --- | --- | --- | --- | --- | --- | --- | --- | --- | --- |
| **Gene** | **Morbid gene** | **OMIM** | **% HI score** | **DDG2P** | **ClinVar** | **Constraint Metrics** | | | |
|  |  |  |  |  |  | **Synonymous (z)** | **Missense (z)** | **LoF (pLI)** | **CNV (z)** |
| *ARHGEF6* | Yes | 300436, Mental retardation, X-linked 46 | 10-20% | **-** | 56dels/56dups/46SNVs | -0.25 | 0.77 | 1 | nan |
| *CD40LG* | Yes | 308230, Immunodeficiency, X-linked, with hyper-IgM | 0-10% | **-** | 59dels/55dups/17SNVs/7ins | 0.82 | 0.92 | 0.86 | nan |
| *BRS3* | no | - | 30-40% | **-** | 52dels/50dups/2ins | -0.49 | 0.90 | 0.89 | nan |
| *FHL1* | Yes | 300696, Emery-Dreifuss muscular dystrophy 6, X-linked; Myopathy, X-linked, with postural muscle atrophy; 300717, Reducing body myopathy, X-linked 1a, severe, infantile or early childhood onset¸300718, Reducing body myopathy, X-linked 1b, with late childhood or adult onset; 300695, Scapuloperoneal myopathy, X-linked dominant | 10-20% | Yes | 58dels/50dups/52SNV/5ins/1indel | 0.59 | 1.29 | 0.92 | nan |
| OMIM: Online Mendelian Inheritance in Man; HI score: Haploinsufficiency Score index - high ranks (e.g. 0-10%) indicate a gene is more likely to exhibit haploinsufficiency, low ranks (e.g. 90-100%) indicate a gene is more likely to NOT exhibit haploinsufficiency (retrieved from Decipher); DDG2P: **Developmental Disorders Genotype-Phenotype Database;** LoF: Loss of function; CNVs: copy number variations; z: Z score is the deviation of observed counts from the expected number for one gene (positive Z scores = gene intolerance to variation, negative Z scores = gene tolerant to variation) (retrieved from ExAC); pLI: probability that a given gene is intolerant of loss-of-function variation (pLI closer to one = more intolerant the gene is to LoF variants, pLI >= 0.9 is extremely LoF intolerant) (retrieved from ExAC); del – deletion; dup – duplication; SNV – single nucleotide variant; ins – insertion; indel – insertion/deletion. | | | | | | | | | |

**BIBLIOGRAPHY**

1. Wechsler D. Wechsler Intelligence Scale for Children—Third Edition. The Psychological Corporation; 1991.

2. Sparrow S, Balla D, Cicchetti D. The Vineland Adaptive Behavior Scales: Interview edition, survey. In: Major psychological assessment instruments. 1984. p. 199–231.

3. Wechsler D. Wechsler intelligence scale for children – Fourth edition (WISC-IV) administration and scoring manual. 2003b, San Antonio, TX Psychol Corp.

4. Jordan VK, Zaveri HP, Scott DA. 1P36 Deletion Syndrome: an Update. Appl Clin Genet [Internet]. 2015;8:189–200. Available from: http://www.pubmedcentral.nih.gov/articlerender.fcgi?artid=4555966&tool=pmcentrez&rendertype=abstract

5. Firth H V., Richards SM, Bevan AP, Clayton S, Corpas M, Rajan D, et al. DECIPHER: Database of Chromosomal Imbalance and Phenotype in Humans Using Ensembl Resources. Am J Hum Genet [Internet]. 2009;84(4):524–33. Available from: http://dx.doi.org/10.1016/j.ajhg.2009.03.010

6. Huang N, Lee I, Marcotte EM, Hurles ME. Characterising and predicting haploinsufficiency in the human genome. PLoS Genet. 2010;6(10):1–11.

7. Vilhais-Neto GC, Maruhashi M, Smith KT, Vasseur-Cognet M, Peterson AS, Workman JL, et al. Rere controls retinoic acid signalling and somite bilateral symmetry. Nature. 2010;463(18):953–7.

8. Kim BJ, Zaveri HP, Shchelochkov OA, Yu Z, Seymour ML, Oghalai JS, et al. An Allelic Series of Mice Reveals a Role for RERE in the Development of Multiple Organs Affected in Chromosome 1p36 Deletions. PLoS Med. 2013;8(2):e57460.

9. Fregeau B, Kim BJ, Hernández-García A, Jordan VK, Cho MT, Schnur RE, et al. De Novo Mutations of RERE Cause a Genetic Syndrome with Features that Overlap Those Associated with Proximal 1p36 Deletions. Am J Hum Genet. 2016;98(5):963–70.

10. Baynam G, Overkov A, Davis M, Mina K, Schofield L, Allcock R, et al. A germline MTOR mutation in Aboriginal Australian siblings with intellectual disability, dysmorphism, macrocephaly, and small thoraces. Am J Med Genet Part A. 2015;167(7):1659–67.

11. Allen AS, Berkovic SF, Cossette P, Delanty N, Dlugos D, Eichler EE, et al. De novo mutations in the classic epileptic encephalopathies. Nature. 2013;501(7466):217–21.

12. Costa-Mattioli M, Monteggia LM. mTOR complexes in neurodevelopmental and neuropsychiatric disorders. Nat Neurosci. 2013;16(11):1537–43.

13. Verdin H, Baere E De. FOXL2 Impairment in Human Disease. Horm Res Paediatr. 2012;77:2–11.

14. Ferrero GB, Howald C, Micale L, Biamino E, Augello B, Fusco C, et al. An atypical 7q11.23 deletion in a normal IQ Williams-Beuren syndrome patient. Eur J Hum Genet [Internet]. 2010;18(1):33–8. Available from: http://www.pubmedcentral.nih.gov/articlerender.fcgi?artid=2987159&tool=pmcentrez&rendertype=abstract

15. Simões MR, Albuquerque CP, Pinho MS, Pereira M, Seabra-Santos M, Alberto I. Bateria de Avaliação Neuropsicológica de Coimbra (BANC). Cegoc;

16. Mervis CB, Morris CA, Klein-Tasman BP, Bertrand J, Kwitny S, Appelbaum LG, et al. Attentional characteristics of infants and toddlers with Williams syndrome during triadic interactions. Dev Neuropsychol. 2003;23(1–2):243–68.

17. Sheng M, Kim E. The Shank family of scaffold proteins. J Cell Sci. 2000;113:1851–6.

18. Halbedl S, Schoen M, Feiler MS, Boeckers TM, Schmeisser MJ. Shank3 is localized in axons and presynaptic specializations of developing hippocampal neurons and involved in the modulation of NMDA receptor levels at axon terminals. J Neurochem. 2016;137(1):26–32.

19. Phelan K, Mcdermid HE. The 22q13.3 Deletion Syndrome (Phelan-McDermid Syndrome). Mol Syndromol. 2012;2(3–5):186–201.

20. Frye RE, Cox D, Slattery J, Tippett M, Kahler S, Granpeesheh D, et al. Mitochondrial Dysfunction may explain symptom variation in Phelan-McDermid Syndrome. Sci Rep [Internet]. 2016;6(19544):1–12. Available from: http://dx.doi.org/10.1038/srep19544

21. Kleefstra T, Brunner HG, Amiel J, Oudakker AR, Nillesen WM, Magee A, et al. Loss-of-Function Mutations in Euchromatin Histone Methyl Transferase 1 (EHMT1) Cause the 9q34 Subtelomeric Deletion Syndrome. Am J Hum Genet. 2006;79(79):370–7.

22. Schwaibold EMC, Smogavec M, Hobbiebrunken E, Winter L, Zoll B, Burfeind P, et al. Intragenic duplication of EHMT1 gene results in Kleefstra syndrome. Mol Cytogenet. 2014;7(74):3–7.

23. Willemsen M, Beunders G, Callaghan M, de Leeuw N, Nillesen W, Yntema H, et al. Familial Kleefstra syndrome due to maternal somatic mosaicism for interstitial 9q34 . 3 microdeletions. Clin Genet. 2011;80:31–8.

24. Yatsenko SA, Hixson P, Roney EK, Scott DA, Schaaf CP, Ng Y-T, et al. Human subtelomeric copy number gains suggest a DNA replication mechanism for formation: beyond breakage - fusion-bridge for telomere stabilization. Hum Genet. 2012;131(12):1895–910.

25. Rosenthal JA, Chen H, Slepnev VI, Pellegrini L, Salcini EA, Di Fiore PP, et al. The Epsins Define a Family of Proteins That Interact with Components of the Clathrin Coat and Contain a New Protein Module. J Biol Chem. 1999;274(48):33959–65.

26. Tsou J-H, Yang Y-C, Pao P-C, Lin H-C, Huang N-K, Lin S-T, et al. Important Roles of Ring Finger Protein 112 in Embryonic Vascular Development and Brain Functions. Mol Neurobiol. 2016;Mar 7.:doi:10.​1007/​s12035-016-9812-7.

27. Tomoda T, Kim JH, Zhan C, Hatten ME. Role of Unc51.1 and its binding partners in CNS axon outgrowth. Genes Dev. 2004;18(5):541–58.

28. Hopp K, Heyer CM, Hommerding CJ, Henke SA, Sundsbak JL, Patel S, et al. B9D1 is revealed as a novel Meckel syndrome ( MKS ) gene by targeted exon-enriched next-generation sequencing and deletion analysis. Hum Mol Genet. 2011;20(13):2524–34.

29. Romani M, Micalizzi A, Kraoua I, Dotti MT, Cavallin M, Sztriha L, et al. Mutations in B9D1 and MKS1 cause mild Joubert syndrome : expanding the genetic overlap with the lethal ciliopathy Meckel syndrome. Orphanet J Rare Dis. 2014;9(72):1–4.

30. De Laurenzi V, Rogers GR, Hamrock DJ, Marekov LN, Steinert PM, Compton JG, et al. Sjögren–Larsson syndrome is caused by mutations in the fatty aldehyde dehydrogenase gene. Nat Genet. 1996;12:52–7.

31. Neumann SA, Tingley WG, Conklin BR, Shrader CJ, Peet E, Muldoon MF, et al. AKAP10 (I646V) functional polymorphism predicts heart rate and heart rate variability in apparently healthy, middle-aged European-Americans. Psychophysiology. 2009;46(3):466–72.

32. Torkamani A, Bersell K, Jorge B, Bjork Jr RL, Friedman JR, Bloss CS, et al. De Novo KCNB1 Mutations in Epileptic Encephalopathy. Ann Neurol. 2014;76(4):529–40.

33. Sheen VL, Ganesh VS, Topcu M, Sebire G, Bodell A, Hill RS, et al. Mutations in ARFGEF2 implicate vesicle trafficking in neural progenitor proliferation and migration in the human cerebral cortex. Nat Genet. 2004;36(1):69–76.

34. Yilmaz S, Gokben S, Serdaroglu G, Eraslan C, Mancini GM, Tekin H, et al. The expanding phenotypic spectrum of ARFGEF2 gene mutation: Cardiomyopathy and movement disorder. Brain Dev. 2016;38(1):124–7.

35. Banne E, Atawneh O, Henneke M, Brockmann K, Gärtner J, Elpeleg O, et al. West syndrome, microcephaly, grey matter heterotopia and hypoplasia of corpus callosum due to a novel ARFGEF2 mutation. J Med Genet. 2013;50(11):772–5.

36. Prosser SL, O’Regan L, Fry AM. Novel insights into the mechanisms of mitotic spindle assembly by NEK kinases. Mol Cell Oncol. 2016 May;3(3):e1062952.

37. Hirai Y, Tamura M, Otani J, Ishikawa F. NEK6-mediated phosphorylation of human TPP1 regulates telomere length through telomerase recruitment. Genes to Cells Devoted to Mol Cell Mech. 2016 Aug;21(8):874–89.

38. Kyrychenko VO, Nagibin VS, Tumanovska L V., Pashevin DO, Gurianova VL, Moibenko AA, et al. Knockdown of PSMB7 induces autophagy in cardiomyocyte cultures: possible role in endoplasmic reticulum stress. Pathobiol J Immunopathol Mol Cell Biol. 2014;81(1):8–14.

39. Allaire PD, Ritter B, Thomas S, Burman JL, Denisov AY, Legendre-Guillemin V, et al. Connecden , A Novel DENN Domain-Containing Protein of Neuronal Clathrin-Coated Vesicles Functioning in Synaptic Vesicle Endocytosis. J Neurosci. 2006;26(51):13202–12.

40. Junutula JR, Mazie AM De, Peden AA, Ervin KE, Advani RJ, van Dijk SM, et al. Rab14 Is Involved in Membrane Trafficking between the Golgi Complex and Endosomes. Mol Biol Cell. 2004;15:2218–29.

41. Lee C, Iafrate AJ, Brothman AR. Copy number variations and clinical cytogenetic diagnosis of constitutional disorders. Nat Genet. 2007;39(July):48–54.

42. Leung TY, Pooh RK, Wang CC. Classification of pathogenic or benign status of CNVs detected by microarray analysis. Expert Rev Mol Diagn. 2010;10(6):717–21.

43. Miller DT, Adam MP, Aradhya S, Biesecker LG, Brothman AR, Carter NP, et al. Consensus Statement: Chromosomal Microarray Is a First-Tier Clinical Diagnostic Test for Individuals with Developmental Disabilities or Congenital Anomalies. Am J Hum Genet. 2010;86(5):749–64.

44. Kearney HM, Thorland EC, Brown KK, Quintero-Rivera F, South ST. American College of Medical Genetics standards and guidelines for interpretation and reporting of postnatal constitutional copy number variants. Genet Med. 2011;13(7):680–5.

45. Torres F, Barbosa M, Maciel P. Recurrent copy number variations as risk factors for neurodevelopmental disorders: critical overview and analysis of clinical implications. J Med Genet. 2016;53(2):73–90.

46. Hoebeeck J, van der Luijt R, Poppe B, De Smet E, Yigit N, Claes K, et al. Rapid detection of VHL exon deletions using real-time quantitative PCR. Lab Investig. 2005 Jan;85(1):24–33.

47. Bustin SA, Benes V, Garson J, Hellemans J, Huggett J, Kubista M, et al. The MIQE Guidelines: Minimum Information for Publication of Quantitative Real-Time PCR Experiments. Clin Chem. 2009;55(4):611–22.

48. D’haene B, Vandesompele J, Hellemans J. Accurate and objective copy number profiling using real-time quantitative PCR. Methods [Internet]. 2010;50(4):262–70. Available from: http://dx.doi.org/10.1016/j.ymeth.2009.12.007

49. Pfaffl M, Lange I, Daxenberger A, Meyer H. Tissue-specific expression pattern of estrogen receptors (ER): quantification of ER alpha and ER beta mRNA with real-time RT-PCR. APMIS. 2001;109(5):345–55.

50. Taylor S, Wakem M, Dijkman G, Alsarraj M, Nguyen M. A practical approach to RT-qPCR-Publishing data that conform to the MIQE guidelines. Methods [Internet]. 2010;50(4):S1–5. Available from: http://dx.doi.org/10.1016/j.ymeth.2010.01.005

51. Samocha KE, Robinson EB, Sanders SJ, Stevens C, Sabo A, McGrath LM, et al. A framework for the interpretation of de novo mutation in human disease. Nat Genet. 2014;46(9):944–50.
